# Graininess of RGB-Display Space

**DOI:** 10.1177/2041669518803971

**Published:** 2018-10-23

**Authors:** Jan Koenderink, Andrea van Doorn, Karl Gegenfurtner

**Affiliations:** Justus Liebig Universität Giessen, Germany; University of Leuven (KU Leuven), Belgium; Utrecht University, The Netherlands; Justus Liebig Universität Giessen, Germany; Utrecht University, The Netherlands; Justus Liebig Universität Giessen, Germany

**Keywords:** colour, colour space, RGB display space, colour metrics, colour pickers, number of colours, colour symmetries

## Abstract

RGB–display space, that is, the ‘RGB–cube’, was sampled at 3,000 locations, uniformly and randomly distributed. Fifty observers contributed 60 samples each. At each location, participants synthesised a copy of the target, using a generic colour picker. The statistical distributions of errors as a function of location are used to define an overall measure of graininess. A smooth field of interpolated three-dimensional covariance ellipsoids represents an explicit, empirical Riemannian metric. The unit step size is about 20 times larger than the size of the classical MacAdam ellipses. We speculate that this metric might be found useful in various settings involving applications, because it reflects typical fuzziness encountered in generic tasks involving colour patterns such as images. Some of the more obvious applications are discussed.

## Introduction

‘How many colours are there?’ This question is often asked: A Google search for ‘how many colours’ yields numerous hits (on April 9, 2018, 14:40 a total of 533,000 was returned). Our first hit (http://www.rit-mcsl.org/fairchild/WhyIsColor/files/ExamplePage.pdf) suggested 10 million and noticed that this is less than the $16.8\times 10^{6}$ colours addressable on one’s computer display. The suggested answer to ‘How many colours are there in the world’ was ‘infinity’.^1^

Overall, responses range (very roughly) from as few as half a dozen to as many as ∞, with a suggested scientifically founded estimate of about 10^7^ (Davidson & Friede, 1953; Judd & Wyszecki, 1975; Linhares, Pinto, & Nascimento, 2008; MacAdam, 1947; Pointer & Attridge, 1998; Poirson & Wandell, 1990; Schläpfer & Widmer, 1993). Reasons for these differences are that ‘colours’ are (among more) variously interpreted as spectral compositions, 32-bit numbers, prototypical qualities, or triplets of colorimetric coordinates.

For an answer colorimetry (or science) counts mutually nonoverlapping just noticeable difference (jnd) regions in the Schrödinger colour solid (see later) for natural daylight, the count being in the 10^6^ to 10^7^ ballpark (Wyszecki & Stiles, 1967). Such an answer is appropriate when colour tolerancing is the issue but is far outside the range of reasonable estimates of colour gamuts in generic professional use by artists or designers (Quiller, 2002).

Consider a few numbers from such design praxis. The PanTone FHI color guide (Pantone, 2018) has 2,310 samples, whereas the Munsell atlas (Munsell, 1905; Munsell, 1912; Nickerson, 1976) contains 1,600 samples but is already overkill for most uses.

Many of the colour charts that have proved serviceable in various fields, such as ‘Werner’s Nomenclature of colours’ (Syme & Werner, 1814) famously used extensively by Darwin, are much more concise.

Professional pastel collections (pastels cannot be mixed but have to be used as such) contain 100 to 200 items, grey-tone pastel collections about 10 to 12, special collections (say, skin colours for portraitists) would have a few dozen items (Mayer, 1991). Artists are perfectly happy with that.

‘Colour wheels’ used by artists generally tend to have 6 to 48 divisions, 12 perhaps being most common. By exception, the widely used Quiller Colour Wheel (Quiller, 2002) based on commercially available pigments has 68. Remember that Newton (1704) initially saw five colours in the sun’s spectrum and that the rainbow has seven colours in various cultures (Dedrick, 1998).

The Farnsworth (1943) ‘hundred hue test’ suggests that most people might distinguish definitely less than a 100 hues, and perhaps more than 50, let us estimate 75. Then there would be 75/6≈12 colours on a side of the RGB cube, thus $12^{3}=1,728$ in the cube’s volume, about the size of the Munsell atlas.

Photographers consider the Ansel Adams (1949) tonal zone scale (10 greys) somewhat of an overkill, visual artists tend to compose in up to seven tones, two and three being most common.

In many applications, one needs a (not too large) number of categorically different colours. A well-known proposal by Boynton (1989) has a set of 11. There have been attempts to enlarge the set, but experience shows that this is unlikely to ‘work’. It is remarkable enough that 11 (just about) ‘works’, given the conventional estimate of the limited processing capacity of humans (Miller, 1956), which is almost certainly far too optimistic (Gobet & Clarkson, 2004). Boynton’s number is of the order of the maximum number of independent colour terms (Berlin & Kay, 1969; Levinson, 2000; Loreto, Mukherjeeb, & Tria, 2012; Saunders, 2000).

Taking the number of Munsell chips as a provisional (perhaps somewhat high) estimate of the practical number of colours, this is about 6,000 times lower than the number (we used 10^7^) suggested by the jnds (Wyszecki & Stiles, 1967), which again suggests a ‘vagueness’ of about 20 times the jnd size ((10^7^/1600)^1/3^ = 18.4…).

We use ‘vagueness’ in a very general sense, covering sloppiness, fuzziness or indistinctiveness of various kinds, intentional disregard of detail or fine distinction, and so forth. Taken together such effects define a certain pragmatic ‘graininess’ of the RGB cube.

### The Research Problem

Why are such numbers so much lower than the jnds classically known from psychophysics (Torgerson, 1958) would suggest? The obvious reason is that jnds are typically measures of edge contrast thresholds for sharp boundaries between two uniform areas (MacAdam, 1942).

Edge detection methods are somewhat problematic, because the crucial importance of the spatiotemporal modulation used in jnd measurement is well known (Noorlander & Koenderink, 1983).

Pure edge detection may be avoided (Krauskopf & Gegenfurtner, 1992), whereas still avoiding the issue of perceptual qualities. This yields slightly higher jnds, though still numbers that are very high as compared with the estimates from praxis.

In any case, such psychophysical measures are not about *colour* differences in terms of qualities. They are about detecting *any* difference, regardless its phenomenological nature.

In contradistinction to this fundamental psychophysics, in design workflows the colour *as a quality* is very relevant. In selecting a colour for a task, the user picks a colour that matches a memory image, perhaps of a coloured patch seen before, perhaps even immediately seen before, because already present in the work in progress. This is common in painting practice.

The user compares or produces (see later) colours but hardly ever finds any need to judge the detectability of edges between uniform patches, ignoring the colours, as in the edge detection method (MacAdam, 1942; Noorlander & Koenderink, 1983). Moreover, colours are usually seen in complex contexts, instead of uniform adapting fields (as in Krauskopf & Gegenfurtner, 1992).

Artists are certainly interested in ‘edge quality’ (Edges, 2018) but hardly in detectability. The case of ‘edge strength’ for (far) supra-threshold modulations is still a research issue (Koenderink, van Doorn, & Gegenfurtner, 2018; Koenderink, van Doorn, Pinna, & Wagemans, 2016).

If one needs to find a paint to cover an abraded patch on a car body, the jnd criterion is essential. In such a case, it is only the visibility of the effects of the overpainting that determines success: Is the repair detectable or not? The colour as such is irrelevant.

On the other hand, if one desires to select a paint to colour an object so as to match one’s imagery of the desired result, only the particular colour is essential. In that case, the jnds are irrelevant.

These two cases are indeed *categorically distinct*. This has a huge effect on the relevant measure of graininess.

To work out the various numbers related to graininess, one needs to take account of some basic geometrical relations. Notice that the various numbers of pastels quoted above should be understood in terms of *dimensionality* (linear, areal, volumetric) and *metric*. This is a basic consideration in judging the various numbers. Consider some simple back-of-the-envelope estimates.

An RGB monitor with regular gamma 2.2 is designed to offer an (at least very roughly) perceptually uniform parameterisation of display colours (Poynton, 2003). Let the RGB coordinates (range 0–1 say) be specified with a precision, or ‘vagueness’, of ɛ=0.1 to 0.2. Then the number of colours would be 125 to1,000 (volume of the RGB cube, thus ɛ^−3^). The grey axis would have 9 to 17 distinct levels (body diagonal of the RGB cube, thus $\sqrt{3}/ɛ$). There would be 30 to 60 distinct hues (length of a progression of six edges, thus 6/ɛ). The set of tints and shades of a hue would contain 18 to 71 distinct colours (the area of a triangle like black-white-red, thus ${ɛ}^{-2}/\sqrt{2}$).

Thus, such rough guesstimates at least get one immediately in the right ballpark. Notice that the typical instrumental precision of the RGB coordinate is 0.004… (i.e., 1/256, one bit of a byte), about 25 to 50 times better than the 0.1 to 0.2 precision assumed above. The number of display colours that can be specified is about 17 million (256^3^), near the often quoted estimate based on jnds.

The estimate of the ‘vagueness’ typical for colour in design work (apparently about 0.1–0.2) is much (say 20 times) larger than typical jnd estimates. Such large vaguenesses immediately appear in tasks that focus on particular *colours* instead of mere edge detectability. It implies (as noted earlier) that the RGB cube does not have room for more than 125 to 1,000 grains (rough estimate). It would seem a priori likely that the graininess would be nonuniformly distributed over the RGB cube.

Here, we arrive at the goal of the present experiments, which is to obtain empirical estimates of the vagueness in typical design tasks. We are especially interested in the spread over a group of naive users and in the distribution of the vagueness over the space of typical colours provided by a generic computer display. Therefore, all results will be presented in this format, rather than in terms of perhaps more familiar colorimetric systems.

Of course, the display RGB coordinates on a regular display (gamma 2.2, white point D65; Poynton, 2003) are exactly what is of relevance to the practitioner and is most widely used outside vision research proper, the main contender being cmyk in the printing business (Gatter, 2005). In terms of number of users RGB wins hands down, no doubt by several orders of magnitude from contenders such as cie–xyz, cielab, lms, and the like.

The preference of vision research certainly makes good sense from the perspective of physiology but not so much from the perspective of phenomenology and not at all from the perspective of praxis (Smith & Lyons, 1996).

### ‘Colour Pickers’ as Natural Tools to Probe Colour Vagueness

In typical use, colours are either specified purely mentally through numerical coordinates, or they will be synthesised by eye measure using one of the many available ‘colour pickers’ (Foley, Dam, Feiner, & Hughes, 2005; Koenderink, van Doorn, & Ekroll, 2016).

The former method is commonly used in formal graphics programming (Foley et al., 2005), coordinate values are typically selected from a set like $\{0,\frac{1}{4},\frac{1}{2},\frac{3}{4},1\}$ or a subset from that (for technical reasons, they would be specified hexadecimal, or {#00, #40, #80, #C0, #FF}). This allows 125 colours (of which 5 achromatic) to be specified, the (perhaps more common) discretisation {#00, #80, #FF} allows 27 colours (of which 3 achromatic). Thus, ‘orange’ r$\cup \frac{1}{2}$g would be specified as #FF8000 (typically one would add a fourth *opacity* channel so as to pack everything in a single 32-bit word, we skip that here). With some experience, programmers do it on the guts. Notice that it implies a vagueness of 0.25 or 0.5, coarse perhaps, but not too bad for a start, because sufficient for many, perhaps most applications. It depends upon the context. In many cases (like design), fewer may actually be better, in others, it might be considered a (slight) disadvantage. In the latter case, a ‘gradient’ tool might save the day, more hues are rarely necessary in the arts.

The latter method would be used to gain full precision and articulation. Formal design often implies selection from a fixed set, whereas free work will use a colour picker, ideally with some convenient user interface, more likely enforced by the software (application programs such as Adobe ‘Photoshop’; Brundage, 2012 and a great many others).

We have made an extensive study of colour pickers and their user interfaces (Koenderink, van Doorn and Ekroll, 2016). We prefer to speak of ‘colour synthesisers’ rather than ‘colour pickers’, because one *picks* from a discrete set, but *synthesises* items in continuous spaces.

An important finding, that pertains to virtually all interface designs, is that essentially all users arrive near their goal in about 20s, after which they enter an errant state in which they do not get nearer to their target, but apparently randomly ramble in its vicinity. The diameter of the region of this final state in the RGB cube is somewhat idiosyncratic, but generally implies a vagueness of 0.1 to 0.2, essentially the same as that guesstimated above on very general grounds.

### The Structure of This Article

We describe a near (though by no means ‘final’) colour synthesiser. It allows naive observers to approach their target as near as possible in a time as short as possible. We suggest possible future improvements, some inspired by the present experience.

Improvements are always possible, some will mainly improve user experience, whereas others might actually improve final articulation. The present synthesiser already improves significantly over most commercial offerings (Koenderink, van Doorn and Ekroll, 2016). We stick to the original implementation for the sake of coherency of the empirical data. The most relevant properties of this synthesiser are summarily reported.

More importantly, we report on a total of roughly 3,000 settings achieved by about 50 naive users. This allows an admittedly coarse, but nevertheless unique, overview of the distribution of ‘vagueness’ over the entire RGB cube. We mention ‘coarse’, because this implies the equivalent of (approximately) a 14×14×14 sampling $(3000^{1/3}\approx 14.42\ldots )$, or a resolution of 0.07—significantly smaller than the empirical vagueness, but rather larger than the jnd resolution.

All data are presented in terms of display RGB coordinates (white point D65, γ=2.2), which makes sense given the type of applications one might envisage (Poynton, 2003). A conceptual treatment of the RGB system, based on fundamental colorimetry (involving only the colour-matching functions, but not the luminous efficiency function) is presented in Appendix A. It is essentially just a generic RGB display system as implemented on consumer television, smartphone, and laptop screens, (perhaps implicit) reason being that it is the unique optimum design for the display of object colours.

Finally, we present an analysis of the results, compare it with the conventional colorimetric jnd data and relate it to common pragmatic understanding.

The present data are fully irrelevant to colorimetry and industrial colour tolerancing issues, because the data only pertain to colour experiences but fail to address psychophysical jnds (whether common edge detection type, or otherwise) at all. Whereas this may be considered unfortunate, perhaps even unacceptable, to some, there indeed exists a large community interested in perceived colours as perceptual qualities, rather than psychophysical thresholds.

Thus, we present the data, because of their likely relevance to the community dealing with colour on a professional design basis, be it from ergonomic, engineering, or artistic perspectives.

## The Colour Synthesiser

### Methods

#### Colorimetric data for the display

Stimuli were presented on the LCD screen of an Apple MacBook Pro 15″ (mid 2007 model). The colorimetric parameters of the primary cardinal colours are known from a photo-spectrometric calibration.

The display was linearised using the Bergdesign Supercal 1.2.4 method and radiometrically calibrated using a X–Rite ColorMunki Photo spectrophotometer. Photometric data of the display are as follows:

**Table:** 

Red	*x* = 0.5995	*y* = 0.3406	*L* = 68.9
Green	*x* = 0.3259	*y* = 0.5723	*L* = 197.4
Blue	*x* = 0.1534	*y* = 0.1346	*L* = 53.2

Measurements were done using gamma 2.2, and colours are expressed in RGB coordinates with respect to this RGB cube (or, if preferred, ‘display–space’).

Stimuli were presented on a background that was a random mosaic representing the full RGB display gamut (illustrated in a later section, Figure 2). This is convenient for several reasons. It closely approximates standard practice, the display space is at least (very) approximately perceptually uniform, and the results are expressed in terms of RGB cube coordinates that immediately appeal to the generic user. Important from a pragmatic perspective, virtually all generic displays are likely to yield at least very similar results. (Consider that images look pretty much similar on modern display units, this is one issue on which all manufacturers converge.)

**Figure 1.**
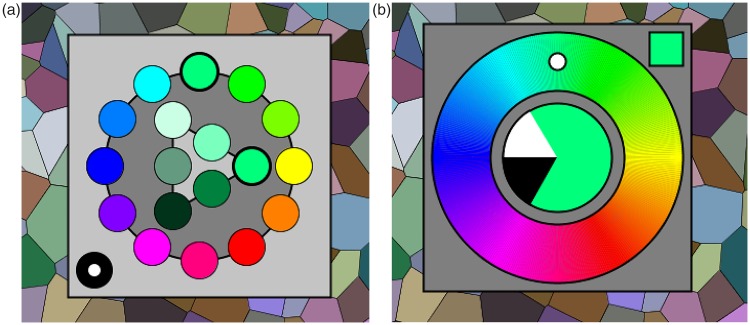
Graphics of the colour-picker in the initial selection phase (left) and the generic adjustment phase (right). In the initial phase, a category is selected by clicking it with the touch pad. In the generic phase, the interaction is by way of the keyboard, the graphics visualises the current state.

**Figure 2.**
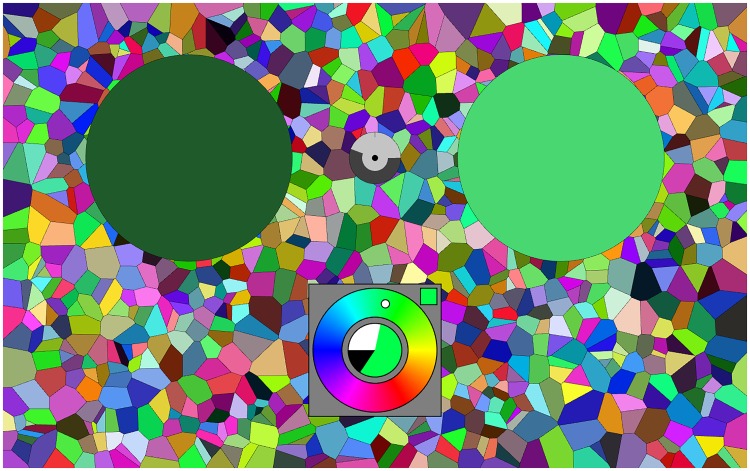
A screen grab from the running experiment. The left disk (dark green) is the target (fixed), the right disk (here a light green) the current synthesis result. The colour picker is at bottom centre, here it is in the final mode, the colour content is large, the white content much less, the black content small. The cursor in the hue circle indicates the present hue (here a cyanish green). The clock is at the centre of the two disks, here it has run for about half the allotted period. The random background is uniformly sampled from the rgb cube. It is refreshed for every trial. The screen is viewed with both eyes, wearing regular corrections, in an otherwise dark room.

Indeed, we look forward to see such results as reported here from a sizeable number of currently popular display units. Changing display conditions such as the nature of the background, or the geometrical layout, are likely to show differences. Our choice of background would be very unusual in a psychophysical setting, it more closely resembles the nature of major applications areas. One might say that the context is ‘generic’, which is indeed our intention.

From a psychophysical perspective such a context has the advantage of offering a *fixed overall adaptation level*. Thus, one cannot expect a ‘Weber fraction’ to be an articulating factor, since the denominator (the adaptation level) is constant throughout the RGB cube. Rather, one expects a central symmetry about the centre of the RGB cube, or—which amounts to the same—opponent pairs like ‘black–white’ (a minus-to-plus-one-scale, say) to be more relevant than a ‘dark–bright’ (a zero-to-infinity scale, say) one.

#### Viewing conditions

Experiments were conducted in a darkened room, thus ensuring a good black level.

The screen was viewed from a distance of about 57 cm. The screen subtended about $32^{\circ }\times 20^{\circ }$. This is not too different from generic use.

When necessary, observers used their natural correction. There is no control over that, but it makes participants feel comfortable, which we consider more important than perfect correction. Differences, if any, are expected to average out.

Viewing was binocular. Again, the main reason is to let participants feel good. The choice most likely makes no difference anyway.

Almost all user interaction was by means of the familiar keyboard. The exceptions were a few seconds at the start of each trial, where a touch pad click was expected.

No participant complained about the interface. This is perhaps not surprising, given the generally less than ideal ergonomic design of common commercial colour pickers (Koenderink, van Doorn and Ekroll, 2016).

#### Participants

Participants were volunteers (PhD students, postdocs, staff) of the Justus Liebig Universität Giessen and the University of Leuven (KU Leuven). They had no colour anomalies as judged by the Ishihara test. Genders include males and females, ages range from 20 s to 70 s.^2^ Our experiment was in agreement with the Helsinki declaration and was approved by the local ethics committee (lek 2013–0018), and all observers provided informed consent.

The bulk of the participants may be denoted ‘naive with respect to the task’, although most no doubt had occasionally used some variety of colour picker before. This implements the kind of ‘general user’ selection we aim for. With a number of participants of about 50, that should indeed work. The randomised trials (see later) guarantee that various differences will tend to average out.

### Design and Implementation of the Colour Synthesiser

To speed up colour synthesis, it was decided to use a two-tiered system. At the initial, very short, stage the user merely selects a general part of colour space. This choice can be undone at the next stage, it is in no way binding, being only introduced to save time in the second stage. A fortunate selection in the first stage puts the participant in the right ball park for the second stage. In the second, more intensive, stage the user adjusts parameters in order to get as close to the target as possible (see Figure 1).

The first stage involves picking a hue from a 12-step colour circle and a tint or shade indication from a 6-point Ostwald-triangle (Ostwald, 1919). This is clearly ‘picking’, so the natural interface is the touch pad. Two clicks suffice.

In the second stage, one needs three-parameter continuous adjustments. Here, the fastest interface uses key board control with visual feedback. We use only the arrow keys. One set (left or right) is used for hue, and the other set (up or down) remains available for a single parameter. This forces a modal design, which is perhaps regrettable.

We use the spacebar to toggle modes between colour, white and black content. The up or down keys always stand for respectively more or less. The actual setting (colour, white and black content, mutually adding to one) is always visible at the centre of the colour circle. The mode is always visible in the square at top-right. As long as participants use their eyes, there should be no confusion. However, it adds a slight cognitive element to the procedure.

In practice, this works quite well, although the modal interface is still less than ideal from an ergonomic perspective. It cannot be circumvented using the keyboard without complicating the interface with additional (to the user arbitrarily assigned) keys. A dedicated interface box would solve this, but the design constraints force us to focus on keyboard and touch pad (or mouse).

Anyway, we find that this colour picker is just as good, if not better, than the best colour pickers we considered in detail in a previous study (Koenderink, van Doorn and Ekroll, 2016). It is quite a bit better than most commercial implementations.

Users are given a verbal explanation and handed a short (one page) manual. They are allowed a few minutes to get used to the interface. Then they are supposed to use it for about an hour, non-stop.

### Display and Task

In principle, the task is a trivial one: There is a target colour that is always visible and at another location, the user needs to synthesise a colour that resembles the target as well as possible (see Figure 2). There is a maximum period to do this, a running clock is provided on the display. When the time is up the next target appears after a short interval, the user may also stop the process at any earlier time, then the next target appears after the same short interval.

The allotted time is such that most users feel to be ready much earlier. However, they never feel assured that their synthesis is the best possible, so many keep adjusting interminably. This is the reason to impose a time limit, it protects participants against vacuous attempts at perfection.

In practice, the nearest approach to the target happens at roughly half the maximum allotted time (see later).

### Empirical Ergonomics

We show only a few results on the empirical ergonomics, because this article concentrates on the distribution of vagueness over the realm of RGB colours. We have complete recordings of over 3,000 attempts at colour synthesis, a huge amount of data. In this article, we mainly consider the *limits* of the tracks. From a cursory investigation, we glean that the full ergonomic results do not deviate from what we have published earlier though.

**Figure 3.**
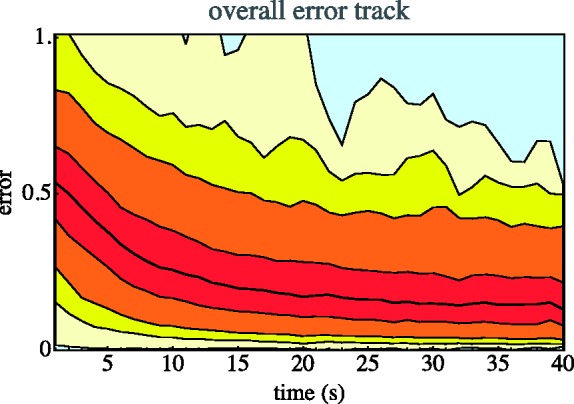
Overall error (Euclidean distance of the result to the target in the rgb cube) as a function of time reckoned from the start of the second stage. We show media, quartiles, 5% and 95% quantiles, 1% and 99% quantiles, and the full range.

The most important ergonomic result is perhaps Figure 3.

Apparently, the synthesis is largely done within about half of the maximum allotted time. Many participants indeed cut that period short. Typically, the synthesis is considered ‘done’ after one or two dozen interface actions.

**Figure 4.**
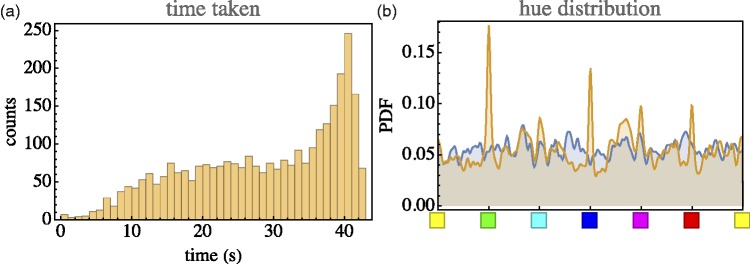
Left: The distribution of time taken per trial. In many cases, the trial was ended before time was up. In some cases, participants persisted until the bitter end; Right: Distribution of hues of target (bluish) and synthesised (orange) result. In the synthesis, one sees that in some cases, a cardinal colour was considered ‘good enough’. This happens for very whitish and (especially) very dark targets. For achromatic colours, the hue is indeterminate.

Consider a few numbers. Time taken (notice that 45 s was allotted) is 31s (median, with an interquartile range of 20–38s). (See Figure 4 left.) Typical number of interface actions is 12 (median, with an interquartile range of 8–17). Of these, more than half are hue adjustments.

**Figure 5.**
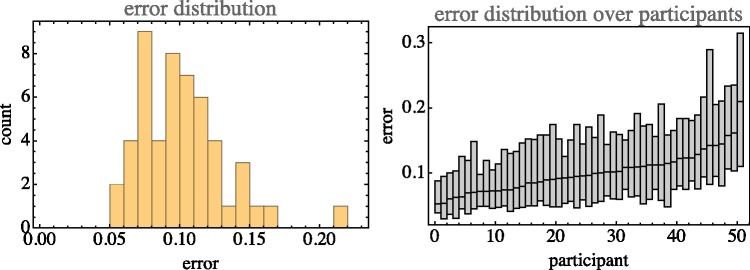
Left: The overall distribution of errors (mean 0.122, standard deviation 0.091). Right: The distribution of errors (median and interquartile range) for all participants, sorted by median.

The median error is 0.098, with an interquartile range of 0.054 to 0.163. (See Figure 5.). In most cases, the closest encounter with the target occurred between 18s and 38s (quartiles, median 26s), about uniformly distributed. At the closest, encounter the distance was 0.073 (median, interquartile range 0.038–0.134), usually the end result was worse.

As shown in Figure 5 right, there is quite a spread of the median final error over the 50 participants. The most accurate participants are twice as good as the least accurate ones.

The distribution of synthesised parameters is not necessarily even qualitatively similar to these of the target parameters (example in Figure 4 right). However, there exist trivial reasons for that. For instance, for very dark shades, the hue is ill defined and the participants find no need to fine-tune it. This makes sense if the error is measured in terms of distance in RGB cube instead of a parameter such as ‘hue’. For near the black point (or, more generally, near the grey axis), the hue becomes increasingly ill defined. A ‘huge’ error as measured by hue can be only a very minor error in terms of RGB location. (At an achromatic point, even the largest hue errors amount to nothing!) Thus, far from taking Figure 4 right as indicating a less than ideal behaviour, one has to interpret it as a sign that the participants are actually doing the right thing.

This evidently tells a story that is very similar to what was found in an extensive investigation (Koenderink, van Doorn and Ekroll, 2016) including several types of colour pickers. The effect seen in Figure 4 right is encountered in a variety of ways, depending on whether a specific colour picker explicitly has hue as an interface parameter. For instance, the effect will be absent if the interface includes just three RGB sliders, whereas it will be highly amplified in colour pickers implemented on the basis of a colour disc with unmarked (achromatic) centre (an unfortunately quite common arrangement).

For any variety of colour picker, users arrive at their setting in about half a minute or less, after that they enter a state of errant adjustments. They continue to perform random walks within about 0.1 to 0.2 (idiosyncratic, also colour dependent) of the target point.

## *Ca.* 3,000 Instances of Colour Synthesis Due to *ca.* 50 Naïve Observers

### Methods

Setup and observers were as described earlier. Indeed, we use exactly the same data set, the difference is only in the definition of the ‘data’.

In the section on ergonomics, we considered the full orbits through display space as the participants manoeuvred to the target. In this section, we consider only their final setting as relative to the target.

### Experiment

Since it was considered possible that cardinal colours might play a singular role and that participants might recognise targets when confronted with another instance of the same, it was decided to draw target colours from a random, uniform distribution over the RGB cube. Thus, there are *no repeats* and *exact* cardinal colours occur with probability zero.

**Figure 6.**
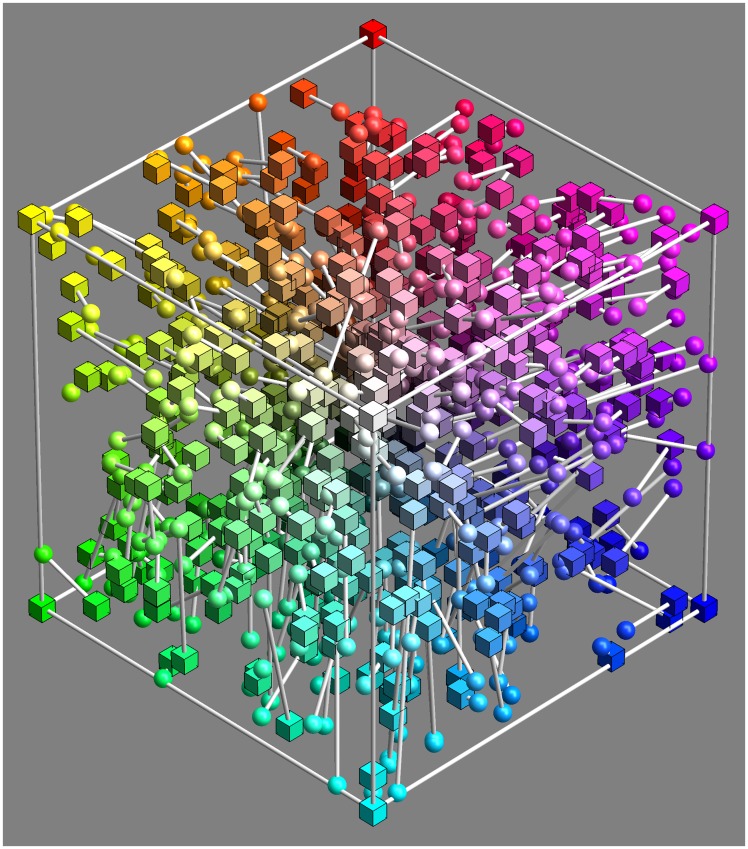
Five hundred randomly selected, raw settings (i.e., less than 17% of the total data volume; the full data set is not shown because too confusing). The spherical marks show the participants approximation to the target indicated as cubical marks. This basic data set is converted to a smoothly interpolated field of covariance ellipsoids. All further analysis is performed on that statistical summary of the raw settings.

Participants simply attempt to synthesise colours as close to target as possible, one after the other (since the targets were random, there are no possible effects of sequence). They simply proceed until a certain time is up (not more than an hour), or a certain number of trials completed (60). Figure 6 shows part of the raw data.

Since the RGB cube volume is so large (due to its three-dimensional [3D] extension), we had to pool data over all observers. This is as planned, because we aim to present generic population data. We managed to collect about 3,000 settings (3,036 precisely), which amounts to a sampling of about 14 per dimension $(3000^{1/3}=14.42\ldots )$.

Doing twice as good would imply eight times $(8=2^{3})$ as many settings, which would far exceed our limited resources in terms of number of participants. The present data set is—for us—the best possible in practice.

We look forward to the date that colleagues will collect even much more extensive data of this kind. We estimate that about fifty thousand or better about a hundred thousand runs would result in a data package that would yield significantly better insight than our initial foray is able to provide.

However, even though indeed limited, the full data set presented here is very large. To describe the results, considerable summarising is required.

The data set is huge, because full tracks through the interface space are provided. It is made publicly available (see acknowledgments). But, although the data contain full records of the actions of users, these are not required for the present purpose. All that are needed are the target colour and the final synthesised colour.

**Figure 7.**
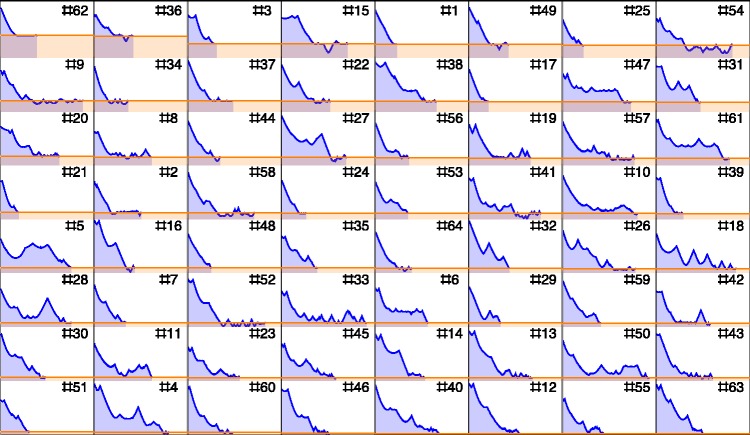
Example of an environment (64 samples) located at rgb = {0.8,0.5,0.2}. For each sample, we show the distance to target as a function of time. Samples have been sorted with respect to the error, from large at top-left to small at bottom-right.

To give at least some (partial) feel for the nature of the full data, consider Figure 7. This does not show the full orbits, but at least the distance to the target as a function of time. It illustrates the nature of the differences between participants quite well. Notice that the worst and the best performers in terms of error both finish in half the allotted period, or less. Some participants that used only a small part of the allotted time did very well in terms of error (Notice no. 39 and no. 51), whereas participants using essentially the full allotted period (like no. 54) do not necessarily do well in terms of error, although some do (like no. 4). Compare participant no. 54 and no.18, the former reaches the final error in a short time, then enters an errant state, whereas the latter keeps approaching the target, albeit with many substantial deviations away from the target.

Of course, a data set based on random location is somewhat harder to analyse than a data set based on a small number of targets with many repeats per target. However, the advantages clearly outweigh the obvious disadvantages, which are mainly of a pragmatic nature.

**Figure 8.**
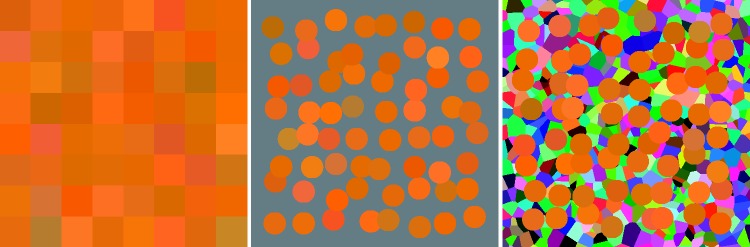
This shows the set of 64 samples in the neighbourhood of a fiducial location at an orange colour. (Notice that the neighbourhood itself is not shown, but only the set of responses.) These synthesised colours are presented as a randomised 8 × 8 array (left), on an average grey background (centre) and on a background randomly sampled from the rgb cube (right). Of course, the set of target colours is expected to show more variability than the set of responses, because the response is reckoned *relative* to the target colour. Even if the responses were perfect, the target colours would still show a spread. In practice, the spread in the corrected responses and the spread of the targets over the region of interest (the region containing the 64 target colours nearest to the orange fiducial location) are not very different, a sign that the neighbourhood size is not too large, but about right in size.

The ‘mismatch’ is the difference between the synthesised and the target colour, each mismatch is a 3D vector. (See Figures 5, 6, 7, and 8.)

Since the target colours are randomly distributed over the RGB cube, the covariance has to be estimated over some finite volume. This volume is determined by the number of data items used to estimate the covariance. We decided on a number of 64 as sufficient for the purpose. With 3,036 samples, the mean radius (Bhattacharyya & Chakrabarti, 2008) of a volume containing 64 samples is $r=(2^{2}/(253\pi ){)}^{1/3}\approx 0.171$. The standard deviation in the radius is small, about 0.007.^3^ Thus, our spatial resolution is about 0.17.

Two (spherical) volumes of radius *r* and centre-separation *d* have an overlap-factor $(d/r-2{)}^{2}(d/r+4)/16$ (Li, 2011). If we sample the RGB cube at 0.1 spacing, d/r≈0.58 and the overlap factor 0.57. The overlap of the samples implies a slight smoothing that allows for effective interpolation functions.

Because we retain all data, there are quite likely to be some ‘outliers’. To handle this, we consider items with *z*-score larger than 3.0 as outliers, with a maximum of 8 per location. After removing outliers, we recompute the covariance and so on, iteratively. Overall, we encounter five outliers (median, with interquartile range 2–8) per location.^4^ Thus, the overall reject fraction is about 7.8%.

All this is done on a uniform cubical grid with space constant 0.1. The sampled covariance matrices are then packaged in a quadratic interpolation function that goes from locations in the RGB cube (3 degrees of freedom) to a 3 × 3 symmetrical matrix (6 degrees of freedom). This is the data structure considered in the remainder of the article. It can be downloaded in the format of a table (see acknowledgment).

### Analysis

#### Basic data-structure

The basic result on which all further analysis is based is the field of covariance matrices that specifies the spatial distribution of the vagueness over the RGB cube. As expected, this distribution is both location and orientation dependent.

**Figure 9.**
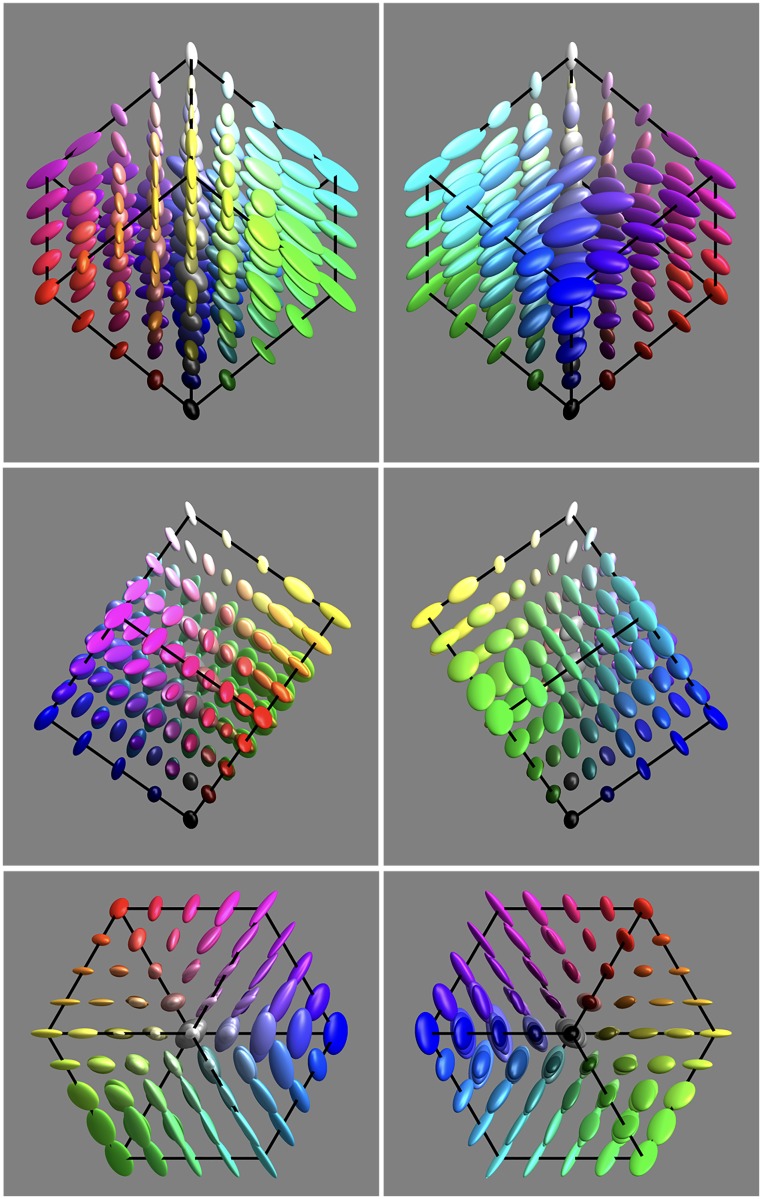
An overview of the covariance field. This represents the raw data of the analysis. It also indicates the grid of fiducial locations.

The basic data are presented in Figure 9 from six different views:

**1st row** orthogonal to the yellow-blue plane, from the yellow and from the blue direction,

**2nd row** orthogonal to the magenta-green plane, from the magenta and from the green direction, and

**3rd row** along the achromatic axis, from the white and from the black direction.

It is evident that the covariance field is smooth, but evidently space variant and that both the spatial attitudes as well as the aspect-ratios of the ellipsoids vary.

It is also evident that the resolution obtained through 3,000 randomly distributed samples is about right. To check this latter point, we redid the analysis using a body-centred-cubic lattice (Hurlbut & Klein, 1985) and sampling the Wigner-Seitz cells (truncated octahedra). In this case, there is no overlap.

The results are not essentially different, perhaps slightly more uneven due to what seems to be random perturbation. In view of this finding, we decided to stick with the data shown in Figure 9. We represent it in terms of a quadratic interpolation function, which is quite convenient, but—if so desired—a new covariance matrix can readily be computed at any point. We use that, for instance, in special cases such as a comparison with the conventional MacAdam jnd ellipses.

#### Number of mutually independent samples

The covariance ellipsoids define local volume elements. Integrated over the RGB cube, one obtains the total number of mutually independent colours (Appendix C). The numerical integration is immediate, the outcome is 1,436 (rounded to integer). Of course, this number is subject to the observational spreads.

The total number of independent colours hides the significant local differences. There are many ways to address this. One way is to consider the variations. The volume elements range over a factor of 40, which implies a factor of 3.4 in linear size. Although this suggests large variation, the volume ratio for the 75% and 25% quartiles is only 2.5 (1.36 in linear size).

Another way to obtain some insight into the spatial variations is to find the number of independent colours in particular submanifolds. In general, we consider line, surface, and volume integrals. This implies restricting the covariance to linelets or planelets, the methods are explained in the section Covariance Ellipses in Planar Sections and Covariance Segments in Linear Stretches in Appendix C.

**Figure 10.**
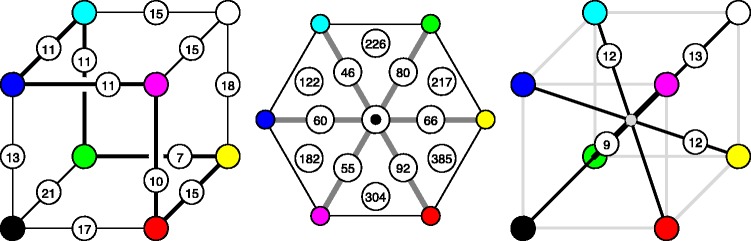
At left, the number of grains along the edges of the rgb cube. Notice the ‘colour circle’ as a nonplanar, regular hexagon. At centre, the number of grains in constant hue triangles (for instance, the white-red-black triangle holds 92) and the number of grains in volume sectors (for instance, the black-white-red-yellow tetrahedron contains 385 grains). At right, the number of grains counted over the (geodesic) body diagonals of the rgb cube. (Notice that the black-white and the green-magenta diagonals partly overlap in this drawing!).

For instance, one may compute the number of colours of a given hue, that are the colours confined to half planes at the achromatic axis. This depends upon the hue. For the six cardinal hues, one obtains the numbers shown in Figure 10 centre. On the average, there are 67 colours in a constant hue triangle, there is a hue dependence by a factor of 2 (1.4 in linear size). To judge these figures,^5^ it is of interest that the grey axis has 13 steps (see later), thus an equilateral triangle with the grey axis as side would subtend about 73 discrete samples.

Of course, one might also approach this issue by counting grains in volume sectors, like the six tetrahedra shown in Figure 10 centre. Here the numbers vary by almost a factor of 3 (i.e., 385/122), reflecting a grain-size variation of $(385/122{)}^{1/3}=1.47$.

Another estimate of interest is the number of colours in a planar section through the centre of the RGB cube, orthogonal to the achromatic axis (shown later in Figure 18; Appendix B). The number is 102, an estimate of the number of samples one would need for a colour disc, the interior of a colour circle.

**Figure 11.**
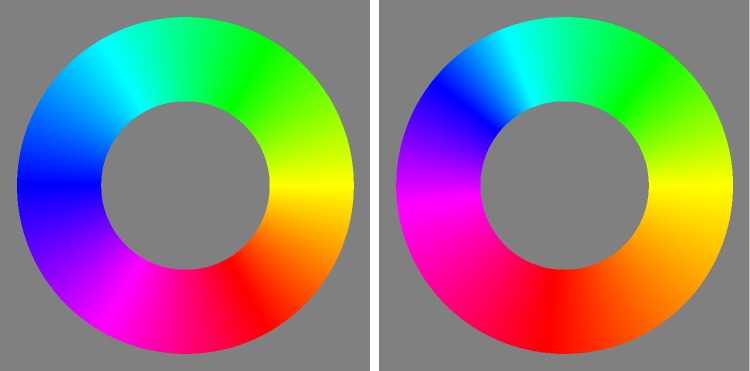
At left, the colour circle equispaced according to the Euclidean distance in the rgb cube. At right, a ‘well-tempered’ scale, graded according to the numbers of grains in equal hue angles.

Instead of constraining the count to planar sections, one may consider counts constrained to linear segments (or curves). Of some interest is the number of independent grey tones over the achromatic axis, for which one finds 13. It is also of interest to count along the locus of optimal colours. One finds the numbers in Figure 10, they make for a total of 59 distinct hues (correcting for double counts). The dramatic changes near the yellow have important implications for the construction of ‘well-tempered colour circles’ (see Figure 11). The well-tempered colour circle (Figure 11 right) is much like the Munsell hue circle.

**Figure 12.**
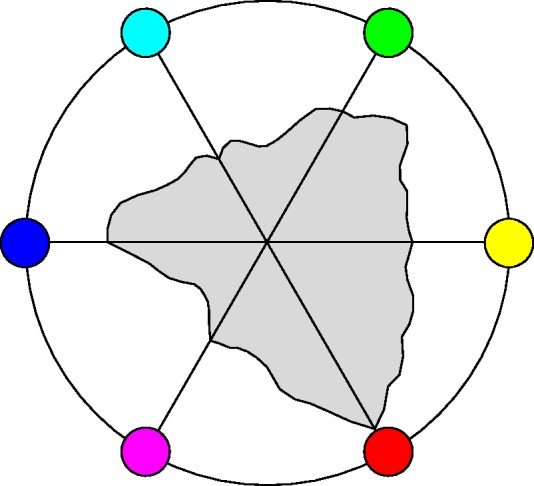
These are distances from points on the full colour locus to the black-white axis. Apparently, the primary colours red, green, and blue are most highly chromatic, the secondary colours cyan, magenta, and yellow least.

Figure 12 plots distances to the grey axis from points on the full colour locus (more precisely, the integrated vagueness from a colour to its projection on the grey axis). One might conclude that the primary colours red, green, and blue are the most chromatic, the secondary colours cyan, magenta, and yellow least, something that appears to be phenomenologically right, at least in the awareness of the authors. Overall, red comes out as the chromatic strongest, magenta as the chromatic weakest cardinal colour. The symmetry (a repeat at 120°) of the plot is striking, although it is certainly not exact.

It is of some interest to consider the degree into which the RGB cube might be considered (very) approximately ‘uniform’. From the results presented so far, it is already obvious that there exist marked nonuniformities and anisotropies. However, it is still of interest to obtain a ballpark notion of the overall structure.

Fairly global measures, like lengths of edges, body, and face diagonals, areas of constant hue triangles, volumes of cardinal hue sectors, and so forth, are of interest in judging whether RGB is roughly fit as a frame, or skeleton structure, of object colour space. This is evidently different from more conventional studies of colour metrics, more ‘primitive’ perhaps, but because of that likely to be of interest to users in visual art and design.

Counting along straight line intervals in general overestimates the separation of the endpoints, because the shortest connection is a geodesic, which is in general curved. Thus, a more correct count will integrate steps along geodesic curves (see Appendix D). Some results for the body-diagonals of the RGB cube are shown in Figure 10. The body-diagonals differ by up to factor of 1.4 in geodesic length (green-magenta 9 compared with black-white 13), thus are not too dissimilar.

The same goes for the ‘medians’ connecting mid-points of mutually parallel faces. One finds 5.5, 7.3, and 6.4. The ratio of the median geodesic length of the body diagonals to that of these medians is very close to $\sqrt{3}$, that is, the Euclidean ratio.

The geodesic distances from the centre of the cube to the vertices have a median of 5.5 and range from 4 to 7. The median of these distances is 0.48 times that of the median of the body diagonals, much as expected by the Euclidean geometry.

**Figure 13.**
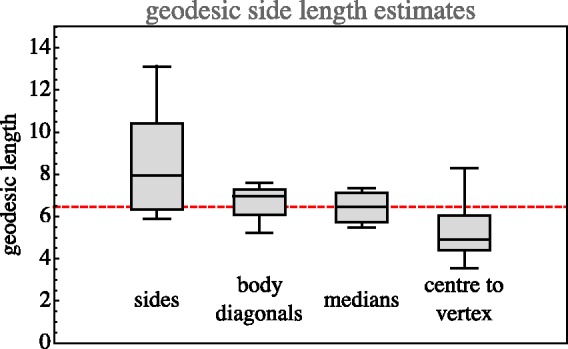
Estimates of the geodesic edge length from various geodesic distances through the rgb cube. (Notice that the cube has 12 edges, 4 body diagonals, 3-medians (face-centre to opposite face-centre) and 8 centre to vertex distances. Here, we present quartiles and total ranges.) The values for the body diagonals have been scaled by $1/\sqrt{3}$ and of the centre to vertex by $2/\sqrt{3}$. The overall median geodesic edge length is 6.5, suggesting a vagueness of 0.15.

As shown in Figure 13, various geodesic distances between mutually far apart locations in the RGB cube correspond quite well. These data also suggest that although the metric is apparently not entirely uniform, it is certainly not wildly nonuniform.

This is also evident from the distribution of the density of the ‘number of colours’. (The density is the reciprocal of the volumes of the covariance ellipsoids.) Over the grid of fiducial locations, the ratio of the standard deviation to the mean is about 62%.

**Figure 14.**
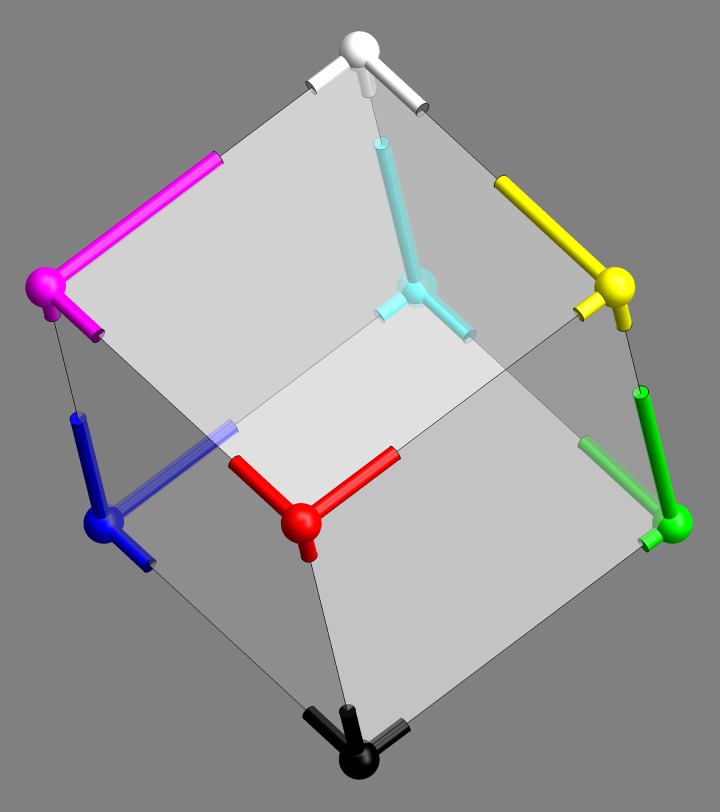
Standard deviation (the covariance ellipsoids confined to an edge, see the section Volumetric, Planar, and Linear Measures in Appendix C) from vertices in the directions of adjacent vertices. Notice that these have been scaled by a factor of 4! Each cylindrical rod indicates a standard deviation at its base point in the direction of its axis.

Nonuniformity can also be shown in Figure 14 (please keep in mind that the indicated standard deviations have been scaled up by a factor of 4!). For instance, notice that the standard deviations from red, green, and blue toward black are much smaller than the standard deviations from cyan, magenta, and yellow toward white. Along the hue locus, the standard deviations at red, green, and blue are much larger than those at cyan, magenta, and yellow.^6^

#### Anisotropies

**Figure 15.**
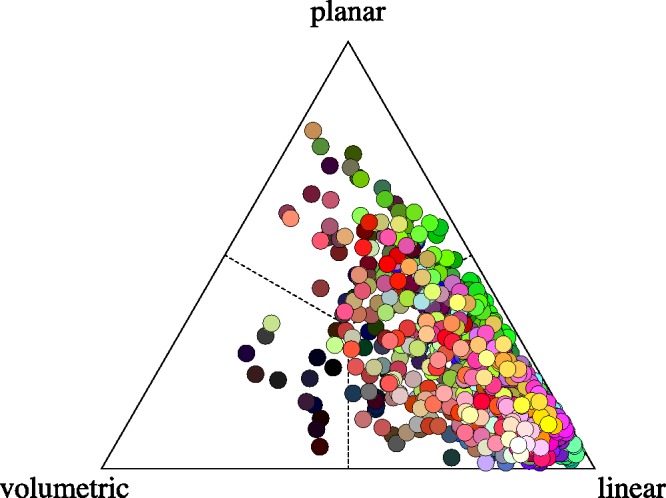
The distribution of anisotropy plotted in barycentric coordinates. The covariance ellipses are predominantly linear.

As a first analysis, one may simply make a scatterplot of all anisotropies in ellipsoid shape space. One finds that the majority of ellipsoids is strongly dominated by the linear part, with only a very minor fraction that would more properly be classified as planar or volumetric (see Figure 15).

Thus, at least in 3D, most ellipsoids are approximately like *rods* (predominantly prolate, or linear). Of course, this may work out very differently in planar sections, remember that a planar section of a broom stick might be circular, it could show about any aspect ratio.

Although indeed mostly rod like, a minor set of ellipsoids is perhaps better described as *coin-like* (or predominantly oblate, or planar) and an even smaller subset as *pea-like* (or predominantly spherical, i.e., volumetric). Again, such differences need not show up in lower dimensional sections, these are 3D (i.e., RGB space) descriptions.

**Figure 16.**
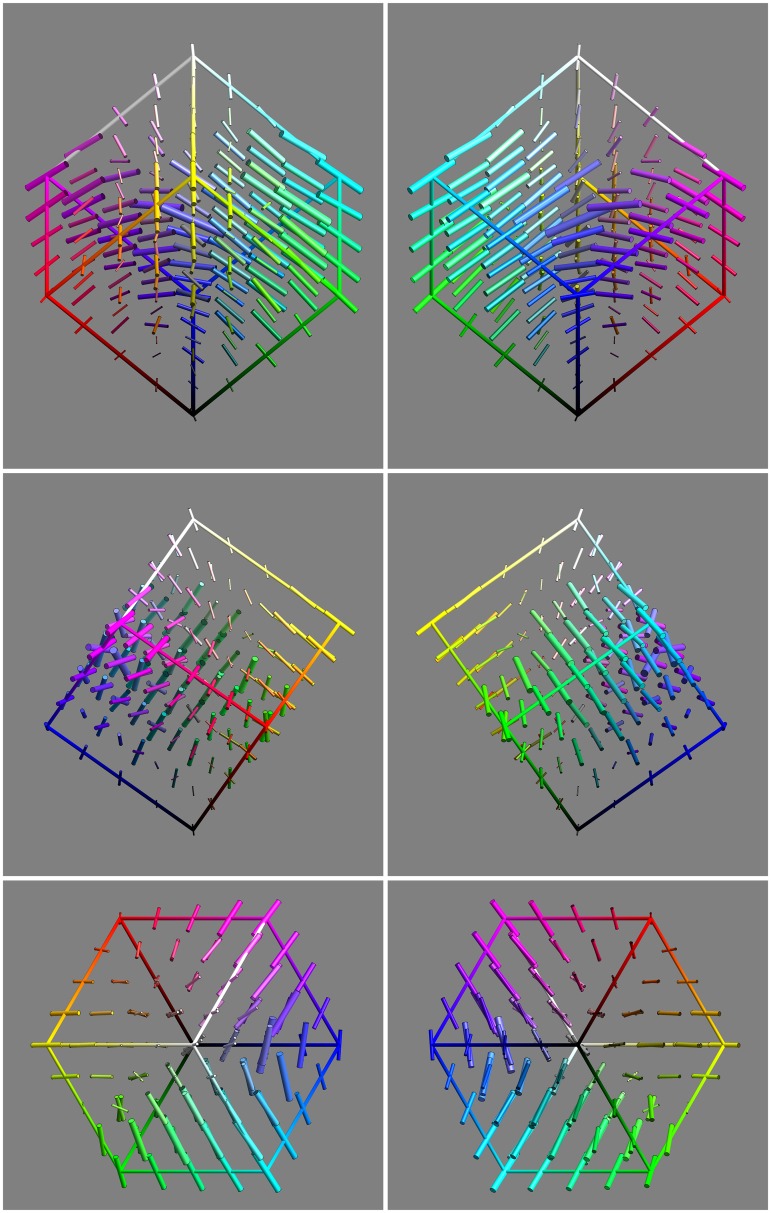
The field of the linear component of the covariance.

The spatial attitudes of the linear parts are far from being uniformly distributed. The linear elements alone go a long way to describe the nature of the field of covariance ellipsoids (see Figure 16).

#### Planar sections through the achromatic axis

**Figure 17.**
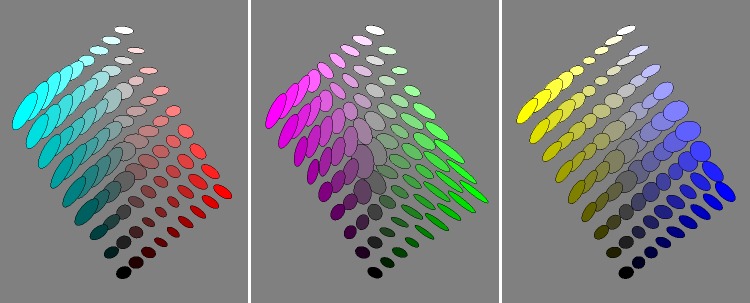
Covariance ellipses in planar sections through the cyan-red, magenta-green, and the yellow-blue planes.

Figure 17 shows planar sections in planes through the achromatic axis. Such sections are similar to the Ostwald triangles of a hue, that are the triangular arrays of mixtures of a ‘full colour’ with white and black, thus displaying the shades and tints of a hue. In the RGB space, the ‘full colours’ are colours with one RGB coordinate equal to 0 and at least one coordinate equal to 1. There exist three such planes that are ‘special’, namely, the red-cyan, the green-magenta, and the blue-yellow planes. The covariance ellipses plotted here are due to the statistical scatter of samples as confined to the planes (thus, the planar ellipses are not just projections of ellipsoids, see the section Covariance Ellipses in Planar Sections and Covariance Segments in Linear Stretches in Appendix C for formal details).

**Figure 18.**
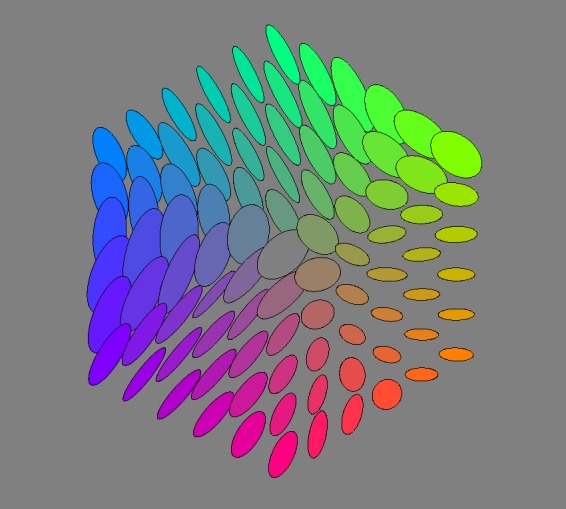
Covariance ellipses in the plane orthogonal to the grey axis, through the centre of the rgb cube.

#### Planar section orthogonal to the achromatic axis at half height

A special plane is the plane r+g+b=3/2. It is evidently orthogonal to the achromatic axis and intersects that axis at half height. This section of the RGB cube is a regular hexagon. Its vertices fail to be full colours (these lie a little above or below the mid-points of the edges of the hexagon, see Appendix B), but this section is the nearest analogon to Newton’s ‘colour circle’, or, rather, colour disk, which renders it interesting.

The planar section is only two dimensional (instead of the 3D rgb cube), thus much easier to intuit. That is also one reason for the popularity of the cie–*xy* chromaticity diagram over XYZ–space. However, the cube section beats the cie chromaticity diagram in terms of visual meaning, because of its symmetry (like Newton’s colour disk) and metric (the chromaticity diagram is part of a projective plane, thus there are no metrical relations displayed).

Figure 18 shows the field of covariance ellipses in this plane. As in the previous subsubsection, the covariance ellipses plotted here are due to the statistical scatter as confined to the plane (see the Covariance Ellipses in Planar Sections and Covariance Segments in Linear Stretches section in Appendix C for formal details).

#### The achromatic axis

The achromatic axis is a special linear section.

**Figure 19.**
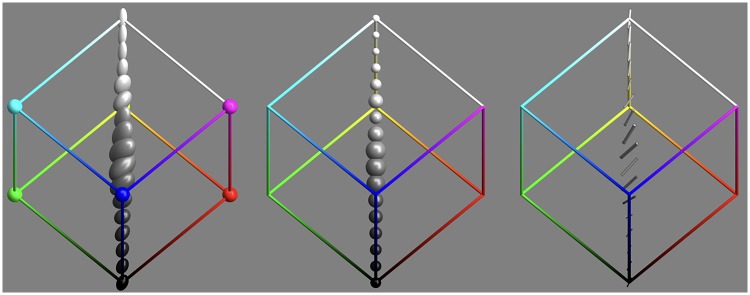
Covariance ellipsoids along the grey axis. From left to right: the covariance ellipses, the volumetric part, and the linear part.

The volumetric and linear parts of the covariance ellipsoids along the achromatic axis are plotted in Figure 19. As noted earlier, there are 13 mutually independent grey levels.

**Figure 20.**
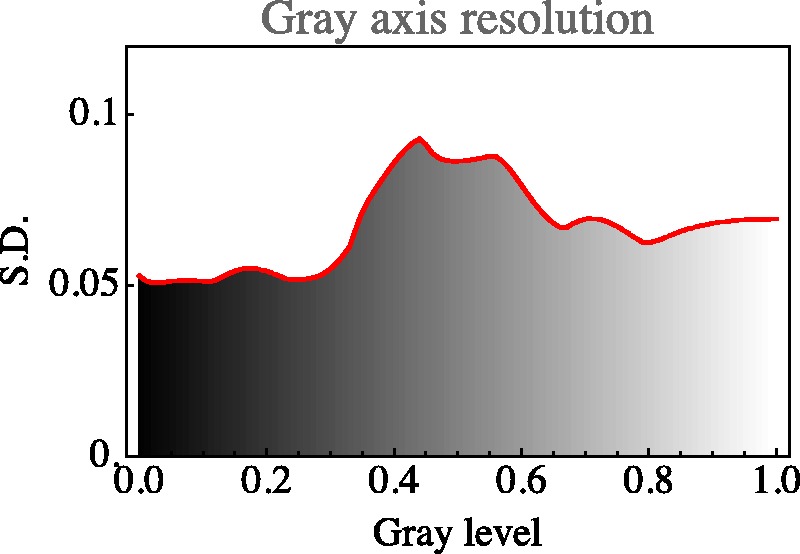
Resolution along the grey axis. Notice how this appears very different from a ‘Weber’s Law’, as it indeed should. Remember that the adaptation level is always the same, which essentially rules out a Weber Law dependence. Furthermore, one has to remember the gamma mapping, which makes a simplistic Weber Law expectation inappropriate. Except from the—perhaps slightly puzzling—central maximum, a nice property of the curve is its symmetry about the grey point. This shows the approximate central symmetry of rgb–display space. Black-white in displays is categorically distinct from dark-light in photometry. In the Hering opponent colour theory—which is likely to be appropriate in the display setting—there is an approximate symmetry about the mid-grey centre.

The distribution over the achromatic axis is far from uniform, the resolution being lowest near the centre of the scale (see Figures 19 and 20).

By eye measure, there is an obvious symmetry under black-white inversion. This will be further addressed in a later section.

#### Symmetries

Colour space is known to admit of various approximate symmetries (Griffin, 2001; Palmer, 1999). A cube has 24 orientation preserving symmetries (the group *S*_4_) and a symmetry order of 48 including rotoreflections. It is perhaps natural to wonder whether some of the symmetries of the cube occur as approximate colour symmetries. Such an idea comes not completely out of the blue, because the habitus of the Schrödinger colour solid is a ‘rounded cube’ (see Appendix A). The colour solid for cie D65 (say) is the region in colour space representing colours of Lambertian surfaces under average daylight illumination, no doubt the gamut that drove the evolution of hominids (Koenderink, 2018).

Indeed, various symmetries of the RGB cube can easily be seen to be present by ‘eye measure’ (especially apparent in Figure 9, say).

To quantify such apparent symmetries, one computes the concordance over a uniformly sampled grid of the covariance matrices and the covariance matrices evaluated at the grid transformed by the symmetry transformation. The correlation measure between covariance ellipsoids is defined in the section Metric in the Manifold of Covariance Matrices in Appendix C, it is perhaps not trivial. Otherwise, computing such correlations simply comes down to numerical integration.

Various measures of concordance, or correlation, are readily available. It most likely is not very important which specific choice is made, but we did not attempt an extensive comparison. A simple measure, that has the advantage of being geometrically meaningful, is the Jaccard index (Jaccard, 1901) for cocentric ellipsoids. It is defined as the ratio of the volume of the overlap to the volume of the union. The Jaccard index is greater than zero and will approach unity for almost fully coinciding ellipsoids. The index is sensitive to size (when one ellipsoid contains the other it is their volume ratio) and shape (the index of two ellipsoids of the same volume depends on the difference of their anisotropies) as well as orientation (the index of two equally shaped, anisotropic ellipsoids depends upon their mutual orientation).

The Jaccard index can readily be constrained in various ways through suitable normalisations of the ellipsoids.^7^ This allows it to be used as essentially a Procrustes metric (Gower, 1975).

Of course, one needs some method that lets one judge a value of concordance as being potentially meaningful. An easy way to do this is to find the concordance for a random scramble of the observed ellipsoid field. Such a concordance is certainly meaningless since a random scrambling does not represent any symmetry at all. One finds that the Jaccard index for the case of a random scrambling is about a third. For typical applications, averaging over about 10^3^ instances, the level is estimated^8^ as 0.320±.005. It is perhaps not superfluous to remark that it is possible for the Jaccard index to drop below the one-third level. This indicates a *discordance*.

There are various symmetries that seem to jump out on first sight. However, we concentrate mainly on two cases that we have considered for other reasons, namely, the grey axis (Figures 19 and 20) and the covariance ellipses in the plane orthogonal to the grey axis, through the centre of the RGB cube (Figure 18).

**Figure 21.**
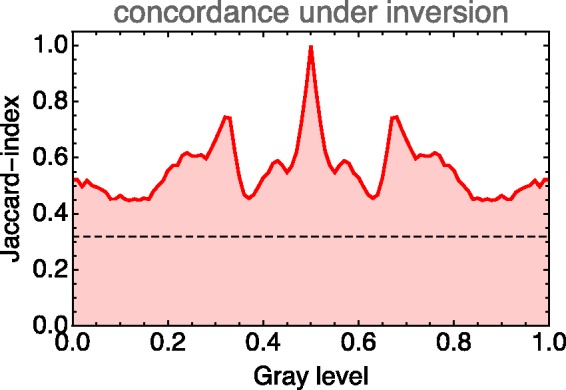
The Jaccard index along the grey axis for the case of inversion about the centre of the rgb cube. The dashed line indicates the level of concordance for a scrambled covariance field.

The case of inversion about the centre of the RGB cube is a basic cubical symmetry. We show its effect on the grey axis in Figure 21. Apparently, there indeed exists such a symmetry along this axis, although it is perhaps not very striking.

In comparison to the grey axis, we present the case of inversion for the full cube. In this case, we average the Jaccard index over a regular sampling grid of a 1,000 (10×10×10) locations. The mean is 0.24, so we have a discordance. This is indeed apparent to eye measure in sections like those in Figure 17.^9^

This comparison (concordance for the grey axis, discordance for the full cube) suggests that the grey axis is very special, something that is immediately obvious from a cursory inspection of Figure 9. The special status of the grey axis is also phenomenologically evident, of course.

There exist six symmetries of the cube that leave the grey axis invariant, namely, the identity, two rotations by ±2π/3 about the grey axis and three reflections about planes that contain the grey axis and either the red and cyan, the green and magenta, or the blue and yellow vertices.

One finds that the rotations about the grey axis yield much larger mean Jaccard indices (both 0.41) than the inversion. This is also true for the reflections. One finds 0.45 (blue and green exchanged), 0.40 (red and blue exchanged), and 0.45 (red and green exchanged).

**Figure 22.**
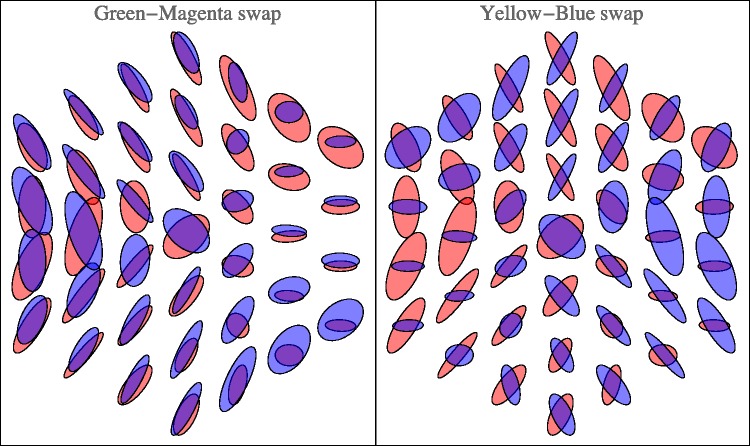
Reflection about the Hering-like axes in the plane at half height. (Here we have slightly redefined Hering’s axes to fit the geometrical symmetry of the rgb cube. ‘Hering–like’ is meaningful, because the axes are evidently perpendicular, showing no effect of a hexagonal symmetry.) These are the ellipses already shown in Figure 18, printed in red and overprinted with a reflected copy printed in blue. Notice that the green-magenta swap indeed yields an approximate symmetry, whereas the yellow-blue swap yields more like an antisymmetry.

**Figure 23.**
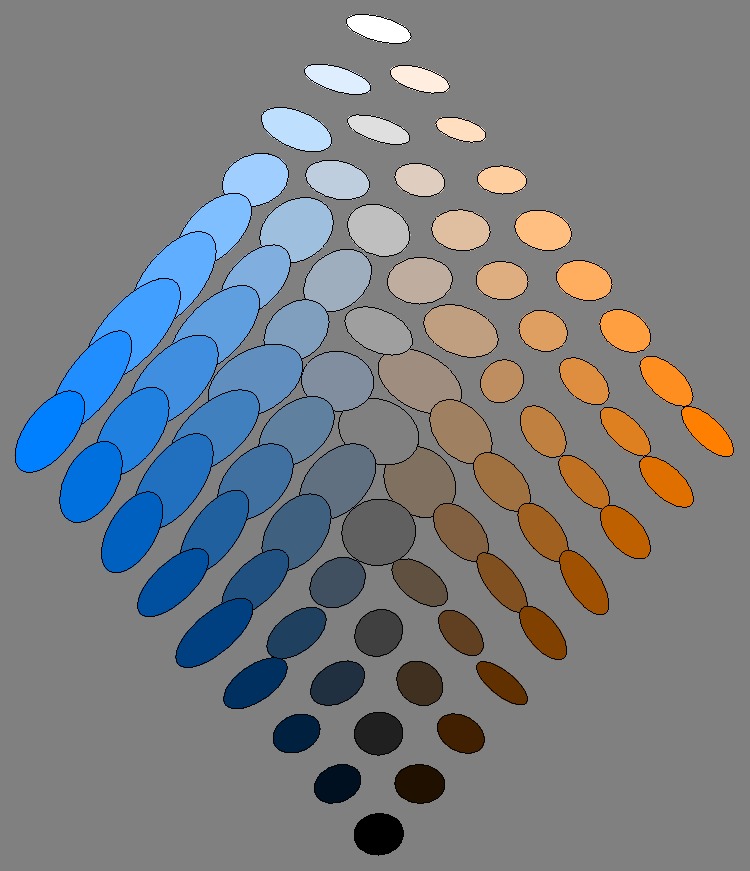
Covariance ellipses in the plane through the grey axis, along the warm-cool direction.

Other reflections in planes through the centre of the RGB cube, not necessarily true symmetries of the cube, are also potentially interesting^10^ (see Appendixes A and B). Figure 18 already gives a vivid impression. The (naturally only approximate) symmetry is perhaps even clearer in Figure 22. Of course, in judging such sections, one should keep other sections in mind. Figure 23 shows the orthogonal section through the cool-warm axis.

This case is especially interesting, because it relates to the possible importance of the Hering axes. Figure 22 lets one judge the results of reflections about the Hering axes in the plane. Apparently, the periodicity is not so much dominated by multiples of $120^{\circ }$, as perhaps expected from cubical symmetry, but rather by multiples of $180^{\circ }$, the Hering axes. This is the phenomenon that strikes one in Figure 18 and is brought out more explicitly in Figure 22. (Notice that one roughly estimates the Jaccard index by eye here!) It is an important observation, we’ll return to it in the discussion.

#### Comparison with the classical MacAdam data

The classical MacAdam ellipses (MacAdam, 1942; Wyszecki & Stiles, 1967) are plotted in the cie-*xy* chromaticity diagram. This is a central projection of colour space, the diagram is not a linear map of colour space, but has the geometrical structure of the projective plane.

To handle this complication, the following procedure was used:

- for a given MacAdam location in colour space, the RGB coordinates are determined;
- if the result lies outside the unit cube, no comparison is possible, otherwise the calculation proceeds;
- an environment of the fiducial is determined in the usual way;
- the points of the environment are mapped to the chromaticity diagram (this implies correcting for gamma);
- a covariance ellipse of these points is determined in the usual way, treating the *xy* coordinates as Cartesian coordinates in a Euclidean metric.

**Figure 24.**
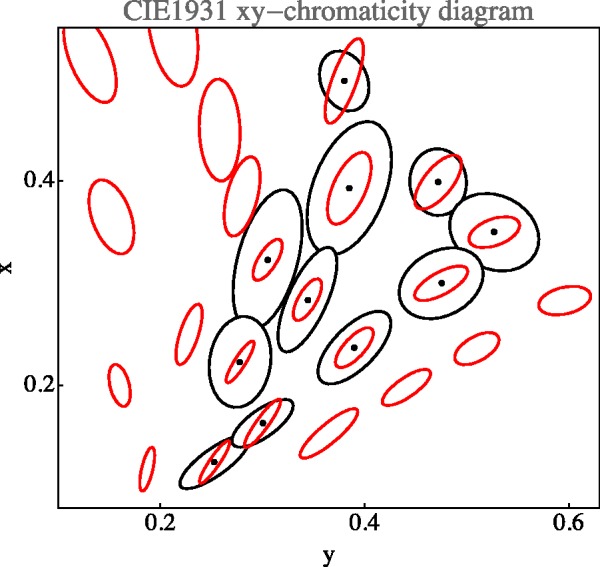
Comparison with MacAdam ellipses. The plot is in the standard cie *xy*-diagram. The MacAdam ellipses (in red) are plotted at 10 times their actual size. The covariances ellipses (in black) were computed from a direct computation in chromaticity coordinates. Notice that many of the MacAdam ellipses fall outside the rgb cube, so a comparison is not possible. These relate to colours that will never appear on generic rgb displays but can be produced in ways that enable concentrating much radiative power in narrow spectral bands, for example, using high power xenon discharges and spectral selections with interference filter-wedges.

Although this procedure is slightly odd from a formal perspective, it perhaps best captures the spirit of MacAdam’s original ellipses. Of the 25 MacAdam fiducials, only 11 lie within the RGB cube. The results of the computations are shown in Figure 24.

The correlation of the ellipsoid shapes (see the section Metric in the Manifold of Covariance Matrices in Appendix C) between the MacAdam ellipses and the empirical data from the present experiment is 0.84. Of course, the sizes of the MacAdam ellipses and the present data are very different. The correlation for the sizes (trace of the covariance matrix) is small.

The ‘size ratio’ may be defined as the square root of the ratio of the traces of the covariance matrices. We find that the size ratio varies from 8.7 to 24.2, with a median value of 15.5 (interquartile range 12.3–19.0). Thus, the ‘vagueness’ as defined earlier is apparently roughly in the range 10 to 20. However, one has to remember that the projective nature of the chromaticity diagram renders the computation somewhat fishy.

Another comparison of some interest is with the data presented by Krauskopf and Gegenfurtner (1992). These authors measured jnd–ellipses using an odd-man-out (4 alternative forced-choice) paradigm. This is pure psychophysics, it does nowhere refer to perceptual qualities, but is very different from the mere edge detection as practiced by MacAdam. A major difference with the present presentation is the context, both (perhaps most importantly) the background and the geometry. These authors quote the number of discriminable colours in an isoluminant plane of their monitor as 2,750, which is 27 times that in the planar section illustrated in our Figure 18. This implies a ‘vagueness’ of a factor of 5.

A factor of 5 is a common kind of ‘safety factor’ used in practice as compared with psychophysics and quite understandable, because of the extreme difference of the nature of the backgrounds.

For those interested in discrimination proper, especially from the perspective of technical colorimetry (as distinct from vision science), a comparison with state of the art colour metrics would perhaps be of more interest. We summarily investigated whether a metric like ciede2000 might ‘explain’ the present data up to some factor. For the global distribution, there is a lack of qualitative concordance. This becomes already evident from a cursory comparison of distances in the RGB cube, like sides, face, and body diagonals (a correlation of all pairwise distances in the RGB cube yields 0.47). Moreover, ciede2000 violates the triangle inequality for green-cyan-magenta, whereas the empirical metric (using geodesic distances) does not, nor does the Euclidean metric of the RGB cube.^11^

We do not draw any conclusions from this as a close match (up to some factor) would have been little less than miraculous. The operational definitions of the grain size are entirely different.

## Discussion

The main result of the experiment is a smooth field of covariance ellipsoids. That is a field of six degrees of freedom (a 3 × 3 symmetrical tensor in 3D, specified by 3 nonnegative eigenvalues and 3 Euler angles, say) at every point in the 3D rgb cube, plotted in Figure 9.

This field encodes a great amount of data, equivalent to about 10^3^ vector-valued samples; thus, 3,000 scalar values, uniformly distributed over the RGB-cube. In the Results section, we already broke this complexity down into a number of partial perspectives.

One aspect, to be considered upfront, is a comparison to the well-known data as canonised and made available by the cie. Unfortunately, it is hardly possible to come up with very detailed comparisons, because of the nature of our data. The statistical errors in colour synthesis reported here are hardly comparable to the conventional jnd data. There are (at least) two reasons for that.

First of all, the task is very different. Second, the background, and thus the current adaptation level is very different.

In the MacAdam-type jnd methods (MacAdam, 1942), the task is to detect the presence or absence of an acute edge between two uniform half fields, embedded in a (often dark) large uniform background. Observers are often instructed specifically to fixate the edge. In the Krauskopf–Gegenfurtner (Krauskopf & Gegenfurtner, 1992) experiments, observers have to make an odd-man-out (simultaneous) decision on four coloured blobs on a uniform background. Observers fixate the centre of the four blobs arranged in a square configuration.

In the synthesis method, the task is to produce a coloured patch such as to appear of the same colour as another, not contiguous coloured patch, both embedded in a background of randomly coloured texture. Observers get no fixation instructions. Indeed, they have to look back and forth between the target and the synthesised patch in order to do the task at all. This setup was designed (as discussed earlier) in order to let the task be similar to cases that occur most often in practice.

Thus, there appears to be no *a priori* reason to assume that the results of these very different tasks will be at all comparable. Indeed, they are not, as apparent from the fact that the typical observational spread differs by more than an order of magnitude.

Classical data as those due to MacAdam are usually plotted in the cie-*xy* chromaticity diagram. This is (from our perspective) very unfortunate, because the chromaticity diagram has the geometrical structure of the projective plane, with the implication that metrical comparisons make no sense. This is indeed a common problem in the literature, where many authors (often silently) treat the cie-*xy* diagram as an affine, or even Euclidean plane. In our comparison, shown in Figure 24, we plot the present data in the chromaticity diagram. Because of the aforementioned problems, we computed the plotted ellipses from data points that were explicitly mapped to the chromaticity diagram first, that is to say, we did not use the interpolated covariance ellipsoids field in the RGB cube. This renders it at least possible to compare relations at a single location. Because we are limited to the display gamut, we can only compare a fraction of the MacAdam ellipses. (About half of the MacAdam data do not apply to generic electronic display units.)

Perhaps surprisingly, given the categorical differences of the tasks, the present data correlate well with the classical MacAdam data (see Figure 24), at least for a comparison of ellipse shapes (correlation coefficient 0.84). The correlation of sizes is insignificant. Of course, there remains the huge gap in magnitude. The linear size ratio is as high as a factor of 9 to 25.

This difference is in the right ball park for our attempt to account for the huge gap between the number of colours in practical representations (as the Munsell atlas) and the number of ‘different colours’ as it is often cited on the basis of jnd data. A linear ratio of 9 to 25 implies a volumetric ratio of about a 1,000 to far over 10,000. If the ‘number of colours’ would be 10^7^ and the number of chips in an atlas 1,500, that would imply a ratio of 19. So magnitudes work out as expected.^12^

Such numbers immediately carry over to the various relevant two- and one-dimensional submanifolds. What remains to be studied is the detailed distribution of magnitude and anisotropy. First, we consider the coarsest features, then we go to more detail.

From Figure 9, it is immediately obvious that the nonuniformities and anisotropies are indeed substantial. It is also immediately evident that they are far from being random. What first strikes the eye is indeed a remarkable degree of symmetry. The symmetries are apparently related to the symmetries of the RGB cube itself. This leads rather directly to two issues:

- perhaps the symmetries are artefacts of the fact that the RGB cube is bounded;
- if not artefact, then the structure of the RGB cube has to be the cause of the symmetries: how come?

We consider both issues.

Are the symmetries artificial? There are various reasons to conclude that this is not the case:

- if boundary effects were a trivial cause, then they should have similar effects everywhere near the boundary. This is evidently not the case at all;
- the symmetries are also present near the centre of the RGB cube where boundary effects are not at all expected.

Indeed, the possibility that such symmetries are artificial can be safely ignored.

Are the symmetries of the RGB cube, considered as a geometrical body, a regular Platonic polyhedron, the *cause* of the observed symmetries of the covariance ellipsoids field? We consider this likely, they are at least an indirect cause.

The RGB cube is the parallelepiped of greatest volume (Appendix A) that can be inscribed into the Schrödinger colour solid for daylight (here cie D65). Thus, it is by no means an arbitrary object, for it is an immediate consequence of the cie colour-matching functions and the daylight spectrum. The symmetries of the RGB cube indeed immediately *derive from* the colour-matching functions and the daylight spectrum, they are in no way *imposed*. We stress this point, because we noticed that it is not generally appreciated.

We propose that the RGB cube is really the appropriate way to represent cie colour space for the case of the daylight illuminant. Since humans evolved under this illuminant, it would seem—from a biological perspective—that the proper description is in terms of a combination of the colour-matching functions (which capture all of physiology that is immediately relevant in this context) and the average daylight illuminant (which captures the generic part of ecological optics in this context). As shown in Appendix A, this also nicely fits the phenomenology, which can hardly be considered a coincidence.

One might say that the RGB cube structure reflects a blend of physiology (as encoded in the colour-matching functions) and ecological optics (as given by the daylight spectrum). Of course, the two can hardly be considered fully independent, as colour vision surely evolved with respect to the needs required by tasks (hunting, foraging, etc*.*) executed under general daylight illumination. It is only the combination of these that counts in terms of biological fitness.

From a historical perspective, this is a formal equivalent of Schopenhauer’s intuitive notion of the colours as ‘parts of daylight’, in which he defined parts of cardinal colours as bipartitions of daylight. This can easily be generalised into tripartitions. Then all cardinal colours, black and white can be mapped on the set of all subsets of the tripartioned spectrum. Indeed, the Hasse diagram of the set of subsets is exactly an RGB cube. Of course, Schopenhauer did not have Schrödinger’s machinery, they published a century apart.

On closer scrutiny, the symmetries are in no way perfect. However, most of the symmetries of the cube are at least approximately present. Reflection about the horizontal axis in Figure 22 yields a visually striking symmetry. (Notice that it may at least partially depend upon the fact that we used the standard display gamma of 2.2.) This symmetry is hardly surprising from the perspective of the generic user (just another ‘fact of life’), but it is perhaps counter intuitive to the physiologist and psycho-physicist.

It is a symmetry that fits very well in Hering’s notions of *Gegenfarben*, where black-white is one of the basic polarities. This would be impossible in a Helmholtz dark-light continuum, which has an entirely different topology. The light-dark dimension is a half line (0…∞), whereas the black-white polarity is the symmetric segment [-1,+1]. This symmetry is also evident from the analysis of the black-white axis (Figure 21).

The overall symmetry is perhaps most strikingly displayed in the ellipsoids drawn in the plane orthogonal to the achromatic axis at half height (Figure 18). The section itself is a regular hexagon, neatly reflecting the basic symmetries of the RGB cube.

From the tripartition of daylight, one might expect to find a hexagonal symmetry of the covariance ellipses field. Then there would be three equally spaced directions and—in terms of Hering’s ideas—three pairs of *Gegenfarben*. Yet, the reality is different (Figures 18 and 22). The pattern is indeed beautifully symmetrical, but mainly a reflection symmetry about the yellow-blue axis, whereas there is perhaps a kind of antisymmetry (in terms of the shape of the ellipses) about a green-magenta axis. Thus, there are only (apart from black-white) two, not three *Gegenfarben*, which is basically Hering’s proposition.

In a recent study, we found the same symmetry in ecological optics (Koenderink, 2018; Koenderink & van Doorn, 2017). The reason is indeed clear.

From the (equi–)partition of daylight perspective, the red, green, and blue ‘parts of daylight’ play fully equivalent roles. This involves a very pure form of trichromacy in that the three parts stand on equal footing. One indeed expects arbitrary permutations of these parts to induce symmetries.

But from the perspective of ecological optics, the red and blue parts border on the infrared and ultraviolet spectral limits, whereas green derives from the mid-part of the spectrum. This implies that orange-blue modulations are due to *spectral slopes* whereas green-magenta modulations are due to *spectral curvatures*. Spectral slopes and curvatures are mutually uncorrelated spectral articulations.

One might distinguish between symmetries that depend on the fact that *in the spectrum* green is between red and blue and those in which red, green, and blue are ‘parts of white’ without further order. Then green is *not* between red and blue. The former structure may be associated with the symmetries noted by Hering, whereas the latter may be associated with Schopenhauer’s notion of ‘parts of white’.

The correlation structure of natural spectra induces Hering-type, not Schopenhauer-type symmetries.

In the arts, these Hering dimensions play an important role. They are often known in terms of the Aristotelian elements (Aristotle, 1982), ‘cool–warm’ and ‘dry–moist’. Especially the cool-warm dimension is used by visual artists on a daily basis, they are also found in affinities to antonyms by naive observers (Albertazzi, Koenderink, & Doorn, 2015); thus, they are apparently deeply ingrained in at least Western culture. However, the underlying cause appears to be of an ecological and ethological nature.

The interplay between the Schopenhauer and the Hering symmetries accounts for the bulk of the symmetries seen in our data. The Schopenhauer symmetries are perhaps best seen in Figure 9 bottom left, where similar $120^{\circ }$ sectors are noticed about the kr, kg, and kb raddii.

The data allow one to increase the efficacy of chromatic scales. For instance, Figure 11 showed the example of a ‘well-tempered’ hue scale. Such nonlinear deformations are likely to be useful in graphics displays where it is desired to use hue as a parameter that is intuitively used by eye measure. One should keep in mind that in such a well-tempered scale (like in the Munsell system), complementaries are not likely to be antipodal (complementaries mutually aligned with the centre), which might be a disadvantage from some perspectives.

Other applications involve the design of tritone scales for scalar (‘grey scale’) data. A popular example is the ‘temperature scale’. We consider it summarily.

**Figure 25.**
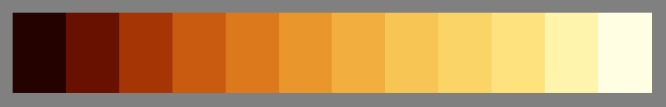
A well-tempered temperature scale of a dozen steps.

The idea is that the edge progression kryw has length 3.0, which is 73.2% longer than the length of the KW axis ($\sqrt{3}$). This edge progression is slightly awkward because of its directional discontinuities. A Bezier curve that starts from black in the red direction and arrives at white from the yellow direction is a smooth alternative that has total length 2.17, still 25% longer than the KW axis. These numbers are Euclidean distances in the RGB cube. Of course, one needs to take the covariance field into account. Then the length of the KW axis is 13, that of the Bezier curve 25, thus a gain of 89%. By dividing the Bezier curve according to the non-Euclidean metric, one obtains a well-tempered temperature scale (Figure 25).

Finally, a remark on the structure of the grey axis (Figure 20). The fact that the resolution is worst near the average grey seems at first surprising. However, metamerism renders average grey rather unstable (as a colour), much more so than either black or white (Koenderink, 2018). Thus, there may well be an evolutionary pressure for the structure here empirically encountered.

## Conclusion

So ‘how many colours are there’ anyway? The exercise described here offers at least a partial answer. The limitations should be kept in mind: We only address ‘object colours’ for a standard daylight illuminant, that is, the interior of the Schrödinger colour solid for that illuminant.

The conventional answer is *millions*, based on jnds, that is, edge detection data. Here, we report *very much* lower numbers, numbers that are in the general ballpark of professional designer’s praxis.

The enormous gap of four orders of magnitude(!) is due to operational definitions. Instead of the conventional edge detection thresholds (jnds), we consider particular colours synthesised by participants so as to visually match given target patches.

The ‘visual match’ is our operational definition of a *particular colour*, the set of ‘particular colours’ being defined as a discrete collection of items that are all mutually distinct, such that their ‘eidolons’ overlap and together exhaust the space of object colours.

A colour counts as an ‘eidolon’ of a fiducial colour if it is not spontaneously seen as a particular colour distinct from the fiducial, say inside a specified probability level from the fiducial as determined by the method of colour synthesis. No doubt, such a match would yield a very obvious edge when compared with the target in the conventional way, for eidolons are very different from metamers.

Not only does it include metamers, but it also includes colours that are many jnds distant from the prototype. Thus, a set of ‘particular colours’ is like a ‘colour chart’, or (say) the (physical) Munsell atlas. In a ‘complete’ colour chart, all samples are obviously distinct, yet close enough that one feels no pressing need for additional ‘in-between’ particular colours.

Here are some of the numbers we arrive at in this study. Of course, one has to understand them with some ‘slop’, the data provided earlier will suggest the kind of slop one might consider appropriate.

- there are just over a 1,000 colours in the RGB cube;
- there are just less than a 100 colours of a given hue, depending on hue;
- there are about a 100 colours in a section orthogonal to the achromatic axis through median grey;
- there are about 60 distinct hues;
- there are about a dozen distinct greys.

Thus, the Munsell atlas might be said to offer ‘about the right’ resolution, albeit representing some oversampling.

‘Just over a thousand colours’ may not sound very impressive, but notice that it still beats the number of greys by a factor of 100. Thus, even a crummy system like this is very effective in helping one *segregate* things by eye. Because of its coarse grains a simple von Kries scheme largely helps fighting effects of metamerism, thus the crummy system is also very effective in helping one *identify*, or find things by eye. From a formal perspective, the dimensionality increase (three instead of one) easily makes up for the coarse graining. (A factor of $1/({ɛ}^{2}\sqrt{3})$ for a grain size of *ɛ*, thus ɛ=0.076… already yields a gain factor of 100, even ɛ=0.24… yields a gain factor of 10.) Thus, such an apparently very coarse system yields an extraordinary boost of biological fitness (Koenderink, 2018).

The size of the covariance ellipsoids (square root of the sum of the eigenvalues) varies only little over the RGB cube, quartiles being 0.09, 0.11, and 0.14, so one may perhaps consider the RGB cube ‘roughly uniform’.^13^

The field of ellipsoids over the RGB cube fits the cube like a glove in that it is not only approximately uniform in resolution but also in that the pattern of (indeed very obvious) anisotropies reflects the symmetries of the cube (see, for instance, Figures 9 and 18) rather nicely. In recent work, we have also found that the metric of the RGB cube does better in predicting a variety of quantitative effects than the far more complicated cie metrics (Koenderink, van Doorn, & Gegenfurtner, 2017; Koenderink et al., 2018).

Thus, the RGB space (based on the Schopenhauer parts of daylight, computed from the cie 1962 colour-matching functions and D65 daylight table, see Appendix A), equipped with the common gamma 2.2, yields indeed a rather ‘natural’ space of display colours, even though its ‘official status’ in the field of colorimetry might be nil.

As the ‘colour for the people’ it is indeed hard to beat and—in terms of raw numbers—already the most common end-user system anyway.

## Figures and Tables

**Figure 26.**
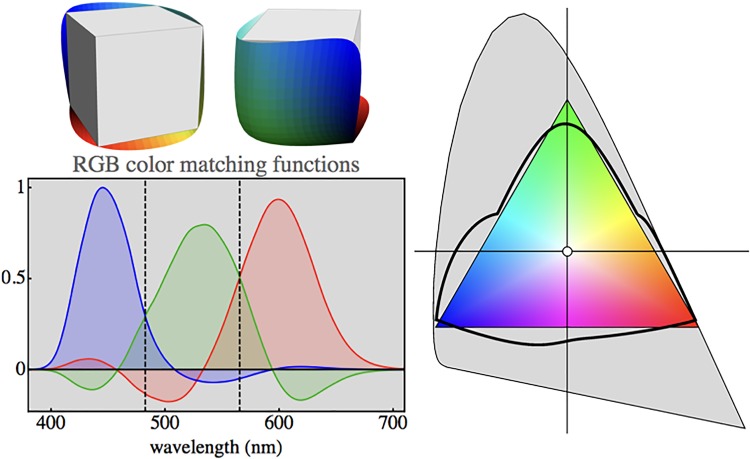
Left-top: The rgb cube in the Schrödinger colour solid (the solid is centrally symmetric, so this shows effectively all). The volume uncovered by the cube contains colours that only very rarely occur in natural gamuts. Left-bottom: The colour-matching functions are narrowly peaked and have minor negative lobes. Their cross-correlations are about −0.24. The dashed lines indicate the cuts for the ‘parts of daylight’. Right: The rgb chromaticity diagram, an equilateral triangle, with the spectrum locus and the Schrödinger full-colour locus. (The layout is similar to that in the cie-xy chromaticity diagram, except that the rgb triangle is made equilateral, thus allowing eye-measure to make at least *some* sense.) The horizontal and vertical axes through the white point turn out to be decisive in the most striking symmetries (Figure 22).

**Figure 27.**
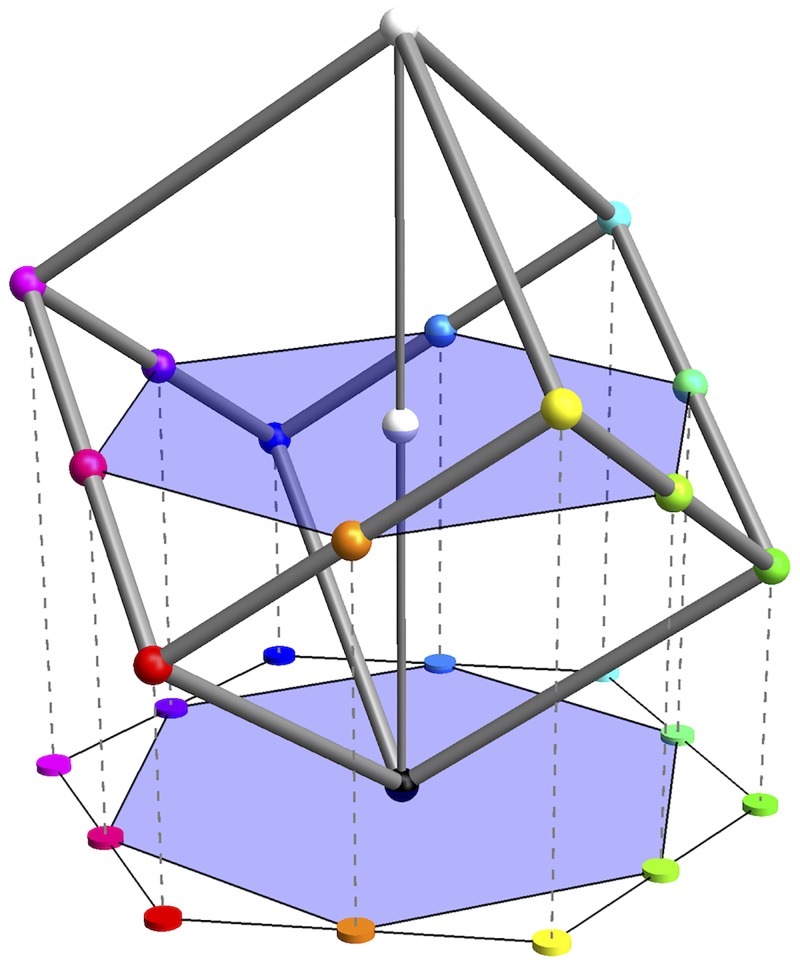
Planar section at half height. The central thin rod is the kw-axis, the thick rods cube edges. The full colours are the edge progression ygcbmry, notice that it goes ‘up and down’, forming a nonplanar, regular hexagon.

**Figure 28.**
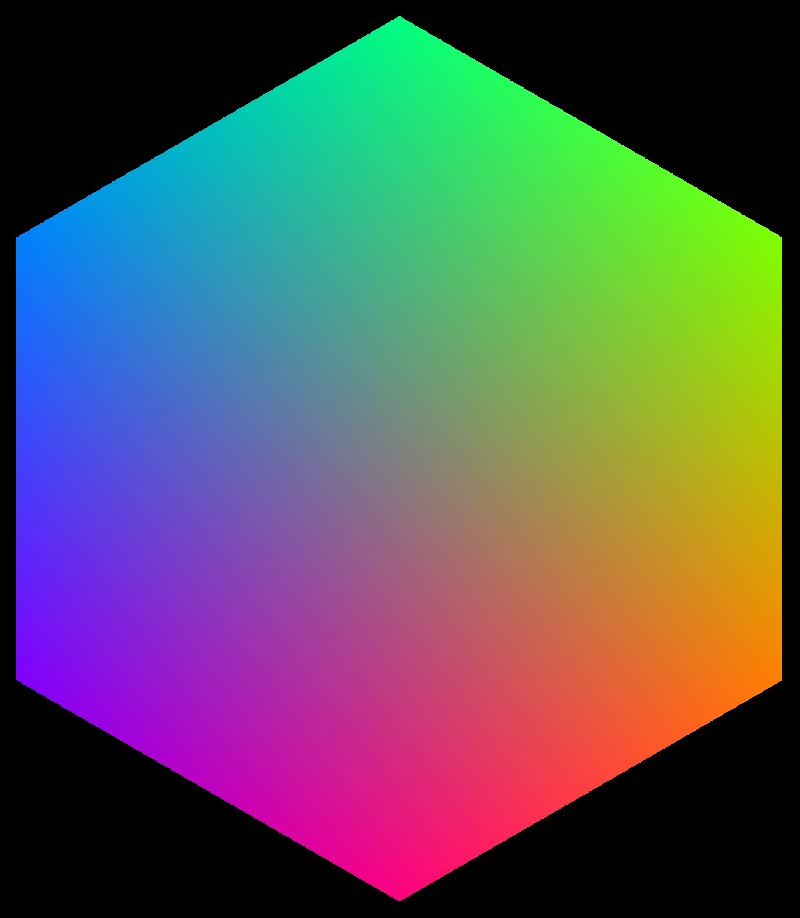
The planar section at half height represents the ‘chromaticities’ (as defined by the orthographic projection illustrated in Figure 27, thus a metric, not a projective plane) in a neat, symmetrical way. In this orientation, the yellow-blue (‘cool–warm’) axis is horizontal, and the purple-green (‘dry–moist’) axis is vertical.

**Figure 29.**
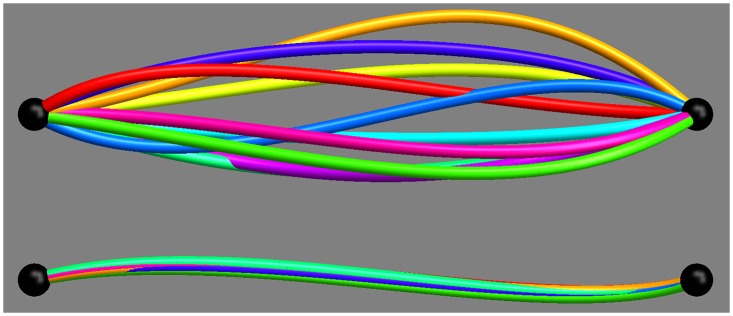
At top, a few random curves connecting two points, at bottom a number of perturbed versions of one such a random curve. The algorithm considers many 10,000 of random curves and considers perturbations of promising ones. This may be supposed to work fine, because the geodesics are expected to be only slightly curved, have little torsion and only minor curvature and torsion gradients. (Colours are random in this figure, they only serve to keep the curves apart.)

**Figure 30.**
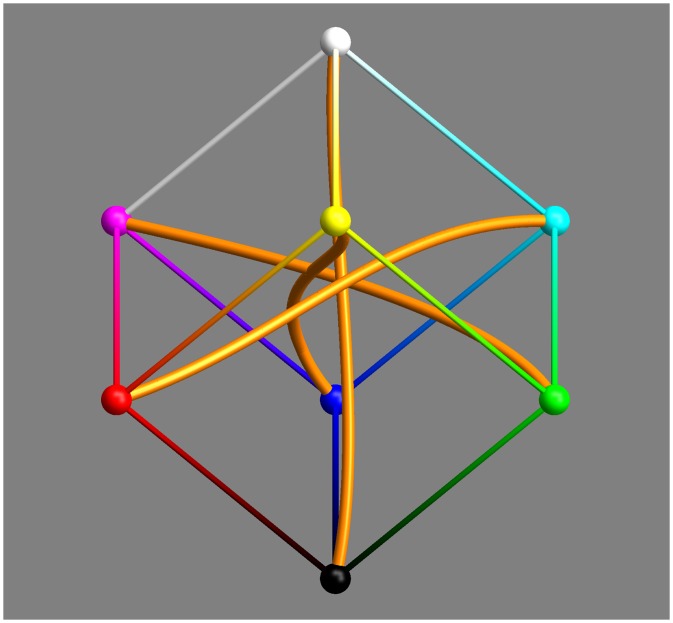
Geodesic body diagonals. The geodesic lengths are kw 26.3, rc 24.1, gm 18.1, and by 24.3. Notice that the geodesic body diagonals fail to meet in a single point, a sign that the Riemann curvature tensor does not vanish identically.

**Figure 31.**
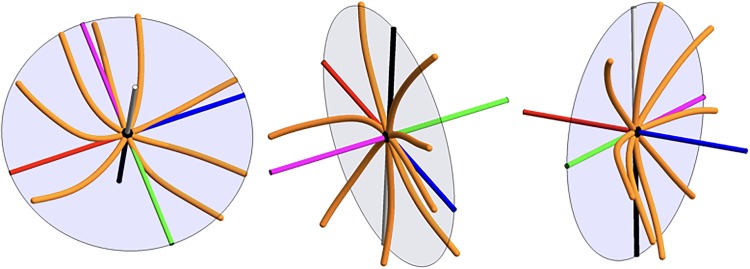
Some geodesics shot out from the centre of the rgb cube. The directions have been constricted to the plane orthogonal to the achromatic axis (left), the red-blue plane (centre), and the green-magenta plane (right). The axes have been indicated by colour (white-black, green-magenta, and red-blue). The Gaussian curvature of the surfaces spanned by the geodesics are components of the Riemann curvature tensor. Notice that some sections are elliptically (right), others hyperbolically (centre) curved.

**Figure 32.**
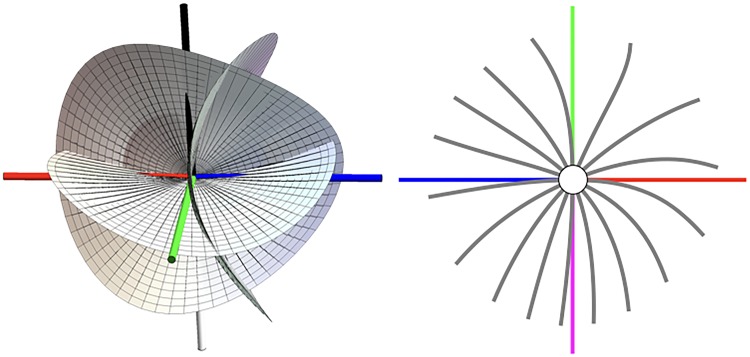
At left, the ‘geodesic planes’ (actually infinitesimal surface patches) at the centre of the cube. They are taken as the surfaces orthogonal to the black-white, red-blue, and green-magenta directions (indicated in colour). At right, some geodesics shot out in the plane orthogonal to the black-white direction and projected upon that plane. Schrödinger hypothesised that such geodesics would be loci of constant subjective hue, but, of course, such a notion can have only heuristic value.

## Notes

1 A reviewer warned us that such a method is potentially biased and certainly nonscientific. We can only agree. So the reader should perhaps try it. We ourselves tried a few more times and remain firmly convinced that our main message is essentially correct. To write a fully documented literature review at this point appears overkill to us.

2 A reviewer of this article objected that this is far from being a representative group of observers. We have to agree. It is hard to say what a representative group would be. Repeating the work with a large variety of groups might give some insights, we are not up to such a task.

3 Thus, the sample size is always the same in terms of numbers (64), but—perhaps remarkably—this implies that the geometrical size of the region is practically fixed too. It is a spherical volume with radius 0.171 ± 0.007.

4 The final means are based on samples in the immediate neighbourhood of a fiducial sample location. In general, we take the 64 samples nearest to the fiducial sample location, but in the averaging process, a few may be marked as ‘outliers’ and removed. The average location of the effective set of samples differs from the fiducial sample location, although it is close. Quartiles of the deviation are {0.024,0.038,0.056}, the distribution is approximately log-normal. From a simulation, we estimate the expected distribution of the ratios of the mean offset to the size of the region. The distributions of the offset of the nearest, the mean and the most distant sample are like this:

The said ratio-distribution is close to a Maxwell distribution with parameter 0.0307. Studying the directions of the 10% largest deviations, one notices a nonrandom ‘drift’, due to the fact that the outliers tend to occur nonrandomly. A clustering yields two major clusters, one positioned near {0.44,0.33,0.78}, with mean deviation {0.044,-0.066,0.047}, the other positioned near {0.33,0.89,0.67}, with mean deviation {-0.11,0.01,0.00}. However, these deviations are small enough that they do not figure in the overall picture (not removing any outliers is not making that much of a difference), so they are not further mentioned in the main text.

5 Of course, one should also remember that we are by no means proposing a *metric* here. For these figures, there is not the least guarantee that the inequality of the triangle will hold, for instance. However, it is not different from such constructions as the cie 2000 colour difference formula, which is not a proper metric either. Such numbers are still useful, when understood in the proper way.

6 This pattern is most likely induced by the gamma 2.2 nonlinear transfer.

7 For instance, one might scale the ellipsoids before comparison so as to have equal volumes, one might even additionally line up their major and minor axes and so forth.

8 This uses the actual field of covariances, but sampled at random pairs of locations, repeated thousands of times.

9 This can be seen in this figure:

Here, the view is like that in Figure 17 right. Evidently, the yellow and blue ellipsoids are at crossed orientations (and remain so after the inversion), leading to a low Jaccard index.

10 In this case, we do not quote an average Jaccard index, because we do not strictly deal with a symmetry of the cube here.

11 Of course, in the case of the ‘Euclidean metric of the RGB–cube’, the gamma is crucial.

12 This is also clear from the overall average covariance ellipsoid. There are various methods that might be used to compute such an average, the simplest is perhaps (*C*_i_ the covariance matrices) $\bar{C}=(\frac{1}{n}\sum_{i=1}^{n}C_{i}^{-1}{)}^{-1},$ which is analogous to the methods used in freshman classes in the case of scalars. The precise method does not make much difference. This is what the average looks like (projections on coordinates-axes-planes added in blue):

The volume is about 1/3,830 of the volume of the full cube (for one standard deviation), thus the ‘number of colours’ works out okay. The principal axes are in the ratio 1:0.70:0.65, whereas the eigenvectors are {0.53,0.24,0.81}, {0.83,0.07,-0.56}, and {0.19,-0.97,0.16}. Notice that the overall mean is not very articulated, it is classified as *volumetric* (the bottom-left quadrangle of Figure 15). Thus, the striking anisotropies seen in Figure 9 and others average out over the full cube. On the average, the covariance structure is close to isotropic.

13 We were severely chastised by a reviewer of this article for saying this. But we remain firmly convinced of the importance of coarse-grained information and rules of thumb. A look out of the window will tell you that the earth is neither flat nor spherical. Yet geographers want us believe in a geoid (a slightly oblate sphere). In our view, the default for the stone age hunter gatherer would be a plane articulated in height, for the early discovers at sea a sphere, for … , and so on. In all cases of practical interest, the simplest rule of thumb that works has priority. Being able to pick the right level of description is vital to your biological fitness.

## Appendix A. The RGB Cube as a Representation of Object Colour Space

Consider the colours of matte (‘Lambertian’), coloured papers normally illuminated with standard daylight. The spectral composition of beams that are scattered to the eye then are conveniently specified by spectral reflection factors, that is the ratio of the scattered radiance of the chip to that of a white chip. Thus, spectral reflection factors are in the range (0, 1), they fill an infinitely dimensional hypercube in the space of spectra. Its projection to human visual space is Schrödinger’s colour solid (Centore, 2017; Koenderink, 2010; Schrödinger, 1920), a centrally symmetric convex volume.

A useful set of colorimetric primaries would be such that the volume of colours with colorimetric coordinates in the range (0, 1) (a parallelepiped) would claim as large a fraction of the colour solid volume as possible. This is a well-defined formal problem. Its unique solution is given by a ‘tripartition of white’, modelled after the bipartitions of white pioneered by the philosopher Arthur Schopenhauer. Schopenhauer (1816) considered himself to be a student of Goethe (1810), albeit with a more comprehensive scientific understanding. He considered colours as ‘parts of daylight’, a more precise notion of Goethe’s original idea of colours as ‘shadows of light’.

The optimum tripartition is easily computed numerically (Koenderink, 2010). The parts have to be Schrödinger optimal colours in the spectral ranges (Figure 26 left) 565 nm–IR (red), 483 to 565 nm (green), and UV–483 nm (blue). Phenomenologically, this is an *equipartition* in the sense that the parts look equally vivid and the unions of RED, GREEN, and BLUE yield vivid CYAN, MAGENTA, and YELLOW, whereas the union of all three yields (by construction) WHITE. The empty set is evidently BLACK. Thus, the set of all subsets of the tripartition of daylight yields all vertices of the RGB cube. It naturally accounts for the nature of (extra-spectral) purple and why the colour circle is closed although the spectrum is not.

In terms of the three parts as primaries, almost all possible coloured papers can be represented by coordinates in the range (0, 1). The RGB cube is inscribed in the Schrödinger colour solid (Figure 26 left), thus there exist colours with coordinate values outside the (0, 1) range, these are nearly Schrödinger optimal colours (Bouma, 1948; Koenderink, 2010).

For databases of natural reflectance spectra, one encounters only a few percent of spectra that cannot be represented and in such cases, the coordinate values are still very close to either zero or one. The reason is that true Schrödinger optimal colours do not occur in nature. Spectral transitions are always gradual, rather than abrupt.

So this optimum RGB—representation is indeed a natural RGB cube that is uniquely implied by standard colorimetry (see Figure 26 right). All that is required to construct it are the cie tables of the colorimetric coordinates of the Standard Observer (Wyszecki & Stiles, 1967; the above calculation uses the cie1962 table) and the cie table for standard daylight (the calculation uses the cie D65 table). The result naturally depends upon the choice of the daylight table. Photometric data on the parts of daylight are (luminance of green set to 100):

**Table:**

Red	*x* = 0.6072	*y* = 0.3928	*L* = 70.0
Green	*x* = 0.2187	*y* = 0.6609	*L* = 100.0
Blue	*x* = 0.1446	*y* = 0.0562	*L* = 12.5

In the CIE-xy chromaticity diagram, the RGB triangle (Figure 26 right) closely approximates the (piecewise curvilinear) locus of Schrödinger–Ostwald full colours (Bouma, 1948; Ostwald, 1919; Schrödinger, 1920) that are optimal colours with mutually complementary transition wavelengths.

The triangle differs somewhat—though not much—from the sRGB triangle (Poynton, 2003), which includes more saturated red, albeit at a cost.

It is only natural that computer displays have evolved to closely resemble an implementation of this ideal RGB cube. It explains why the displays offered by different manufacturers are all so remarkably similar. It also explains why users find the ‘cardinal colours’ defined by the vertices of the RGB cube ‘natural’. These are the ‘primary colours’ (Schopenhauer’s parts of white) R, G, and B, the ‘secondary colours’ c=g∪b, m=b∪r, and y=r∪g, and the achromatic colours k=Ø and w=r∪g∪b.

Here, the operator ‘∪’ signifies set union, as implemented by the superposition of incoherent electromagnetic beams. A scalar multiplier μ in the range (0, 1) signifies physical attenuation (e.g., using a neutral density filter), thus μr for $\mu =\frac{1}{2}$ is a shade of red and r$\cup \frac{1}{2}$g is orange.

An arbitrary colour f can be specified through its coordinates {*r*, *g*, *b*}, thus f=rr∪gg∪bb. Geometrically they are conveniently specified as points in the ‘RGB colour cube’, where the coordinates {*r*, *g*, *b*} are used as Cartesian coordinates defined by the parts of daylight. This recognises the tripartition of daylight as the phenomenologically *equipartition* noted earlier. Thus, r={1,0,0}, g={0,1,0}, b={0,0,1}, and so forth. The geometrical representation will be the preferred one in this article, as it allows us to represent formally complicated relations in an intuitive manner.

All data will necessarily be presented in terms of the RGB values of some *particular* display unit (specified in the Methods section). However, since all display units are very similar and all approximate the ideal prototype, this is actually of generic utility. Just consider that a certain JPEG image may be downloaded by millions of people and displayed on numerous different and typically uncalibrated devices. Yet, only a minor fraction will appear ‘unacceptable’ if the JPEG is any good to begin with. This is only possible because users—and thus the display industry—agree pretty much on what the cardinal colours KW–YGCBMR *look like*.) In any case, full colorimetric data for the display are provided, so data can easily be transformed through standard methods into any representation the reader might fancy.

More importantly, the data are in a format that will immediately be relevant in praxis.

## Appendix B. Geometry of the RGB Cube

The structure of the RGB cube is simple enough (Küppers, 1978, 1989, 2005), but here are a few properties that are sometimes overlooked, or misunderstood.

Notice that we consider a unit cube, thus edge size is 1.0, implying that the face area is 1.0 and the volume of the cube is 1.0. The face diagonal size is $\sqrt{2}\approx 1.414\ldots$, and the body diagonal size is $\sqrt{3}\approx 1.732\ldots$. The shortest edge progression from black to white has length 3.0. The total length of the full colour locus is 6.0. These lengths are used at various places in the text.

Such numbers are often of interest. For instance, the length of the grey axis is 1.7…, since it is a body diagonal, where the length of the edge progression kryw (i.e., the conventual ‘temperature scale’) is 3.0, thus about 1.7 times as long as the grey axis, often quoted as a major point in favour of the temperature scale for display of scalar values. Of course, one really has to express the length in terms of the metric implied by the covariance ellipsoids here.

To aid the intuition, it is useful to imagine the cube with the black-white body diagonal (kw-axis) vertical (Figure 27). This is the standard representation as used in virtually any ‘colour solid’, perhaps starting with Runge’s (1810) ‘color sphere’, present in Ostwald’s (1919) double cone and used in generic images of the Munsell (1905) tree.

Cubes have many symmetries, like reflection planes, that appear irrelevant to colour. Symmetries that are relevant are those that conserve the kw-axis (although perhaps inverting it). These are inversions about the centre, rotations by 2π/3 about the kw-axis and combinations of those, as well as reflections in planes spanned by the kw-axis and edges on k or w.

The planar intersection at half height is a hexagon (Figures 27 and 28). It is used in Figures 18 and 22, because it yields a representation similar to the familiar ‘colour disk’ as originally used by Newton.

In assessing the influence of symmetries we used the RGB cube of display space, not colorimetric space. In the latter case, the black-white inversion would hardly make sense.

## Appendix C. Covariance Ellipsoids

A few properties are used over and over in the text. We succinctly consider them here.

### Covariance Ellipses in Planar Sections and Covariance Segments in Linear Stretches

Given a covariance matrix in RGB space, one often needs the covariance ellipse in a plane, or the covariance segment in a line. Let the volumetric covariance matrix be denoted C and let the corresponding planar covariance matrix in the plane spanned by the unit vectors {*e_i_, e_j_*} be c. Then *c_ij_=e_i_* C *e_j_*.

Likewise, the covariance along the direction of the unit vector f is f C f. In the (common) case that the covariance along a curve is needed one simply uses the tangent vector to the curve.

### Anisotropic Structure of Covariance Ellipsoids

The empirical covariance ellipsoids are general triaxial ellipsoids in all kinds of spatial attitudes. When considering their shapes, the spatial attitude is ignored and one may define the shape by the ratio of eigenvalues (say ordered from largest to smallest) $\lambda_{1}:\lambda_{2}:\lambda_{3}$.

It is of interest to consider the ellipsoids as made up of a volumetric (spherical), planar (planar ellipse), and linear (line segment) part. A natural definition is $\{V,P,L\}=\{\lambda_{3},\lambda_{2}-\lambda_{3},\lambda_{1}-\lambda_{2}\}/\lambda_{1}$, where V+P+L=1, thus {V,P,L} may be considered barycentric coordinates in ellipsoid ‘shape space’.

### Volumetric, Planar, and Linear Measures

Consider a covariance matrix with eigenvalues $\left\{\lambda_{1},\lambda_{2},\lambda_{3}\right\}$, then we define its volume as $V(r,g,b)=\frac{4}{3}\pi \sqrt{\lambda_{1}\lambda_{2}\lambda_{3}}$. The number of elements in a given subvolume Ω of RGB space is found as $\int_{\Omega }(1/V(r,g,b)\text{d}v$.

Next consider a planar covariance matrix with eigenvalues $\left\{\lambda_{1},\lambda_{2}\right\}$, then we define its area as $A(r,g,b)=\pi \sqrt{\lambda_{1}\lambda_{2}}$. This area depends upon the attitude of the planar element too. The number of elements in a given surface region Ω in RGB space is found as $\int_{\Omega }(1/A(r,g,b)\text{d}a$.

Finally, consider a linear covariance with variance *λ*, then we define its length as $L(r,g,b)=2\sqrt{\lambda }$. This length depends also on the orientation of the linear element. The number of elements in a given stretch of curve Ω in RGB space is found as $\int_{\Omega }(1/L(r,g,b)\text{d}\ell$.

### Metric in the Manifold of Covariance Matrices

The structure of fields of covariance ellipsoids has been the topic of extensive research in recent years (Dryden, Koloydenk, & Zhou, 2009). We only partially profit from this, intentionally focussing on well-established classical methods.

The Euclidean norm of a matrix *S* (the Frobenius norm; Golub & Van Loan, 1996) is given by $||S||=\sqrt{\text{Tr}S^{T}S}$. The mutual distance of two matrices *S*_1_, *S*_2_ is defined as $d(S_{1},S_{2})=||S_{1}-S_{2}||$. In many cases, it may be useful to consider the distance of functions of a matrix, such as the logarithm or the square root.

One defines $f(S)=\mathrm{Vf}(\Lambda )V^{-1}$, where $S=V\Lambda V^{-1}$ denotes the eigensystem representation (Λ the diagonal matrix of eigenvalues) of *S* and f() the (scalar) function. In the case of covariance matrices, all eigenvalues are nonnegative—generically positive—making the algebra easy.

Various alternatives are possible and various make indeed good sense (for instance the distance based on Cholesky decomposition) in the present application, but we stick with the simplest case in this study.

We also need to define the mutual correlation of two matrices *S*_1_, *S*_2_. A suitable definition is $\text{Tr}\frac{1}{2}(S_{1}S_{2}+S_{2}S_{1})/\sqrt{\left||S_{1}||||S_{2}|\right|}$. For a field of covariance matrices, one averages over the space through appropriate integration.

## Appendix D. Geodesics

Because the metric is not analytically specified, numerical methods are needed to construct geodesics by numerical integration of empirical data. Because of that, we consider two cases that are conceptually not different, but call for very different methods of calculation. One case involves geodesic connections between two given points. Such connections are needed in order to find the geodesic distance between two points. The other case involves ‘progressive geodesics’, that are geodesic orbits that depart from a given point in a given direction and are prolonged for some arbitrary distance. Due to the accumulation of errors, the maximum geodesic distance that makes some sense is limited. Such geodesics are mainly of interest to study the shape of the geodesics in the neighbourhood of a point.

### Geodesic Connections

Given two points, one considers the lengths of all connecting curves. The geodesic is the one of minimum length. In our application, there is no need to worry about possible multiple solutions.

We implemented a ‘harmony seeking’ algorithm (Geem, Kim, & Loganathan, 2001) in order to find an approximation of the geodesic (Figures 29 and 30). In the case of a Euclidean space, this algorithm comes up with a straight line segment and a close approximation to the Euclidean distance (better than $10^{-6}$ relative error in a few minutes of computation). One needs to search through huge collections of random connecting curves and slightly perturbed deformations of them. Random curves and perturbations of such are easily constructed by generating random control points of Bezier curves. The algorithm is simple enough to implement, but the process is very computationally intensive. On a regular laptop (ca. 2016 or later generation), it is a matter of minutes to reach 1% accuracy in the geodesic distance between any pair of points.

### Progressive Geodesic Orbits

Progressive geodesics can be computed by integration of the geodesic equation (Jürgen, 2002). This requires one to find numerical approximations to the Christoffel symbols and their derivatives. This is conveniently done by first constructing an interpolation function for the empirical covariance matrices.

We use the covariance matrix to define the metric tensor, conventionally denoted *g_ij_*.

One proceeds by computing the Christoffel symbols of the first kind $\Gamma_{\mathrm{kij}}=\frac{1}{2}(g_{\mathrm{ki},j}+g_{\mathrm{kj},i}-g_{\mathrm{ij},k})$, where the notation ‘*j*’ denotes $\partial /\partial x^{j}$, a partial differentiation with respect to a coordinate. The differentiation has to be done numerically; in Mathematica we simply differentiate the interpolation function.

The Christoffel symbols of the second kind are defined as $\Gamma_{\mathrm{ij}}^{m}=g^{\mathrm{mk}}\Gamma_{\mathrm{kij}}$, where we use the conventional Einstein summation convention. The Christoffel symbols satisfy the symmetry conditions $\Gamma_{\mathrm{kij}}=\Gamma_{\mathrm{kji}}$, or $\Gamma_{\mathrm{jk}}^{i}=\Gamma_{\mathrm{kj}}^{i}$, which implies that the space is torsion free.

This is all that is needed to compute the progressive geodesic from a point in the direction of the vector *v^i^* (‘initial speed’). The Taylor expansion is (remember the summation convention (here over the indices *j* and *k*), the three components of *X*(*t*) are labelled by the index *i*):

(1) $$X(t{)}^{i}=tv^{i}-\frac{t^{2}}{2}\Gamma_{\mathrm{jk}}^{i}v^{j}v^{k}+O(t^{3})$$

We use this equation iteratively in order to construct the geodesic (Figures 31 and 32).

To proceed and construct the Riemann curvature tensor, one needs yet another differentiation (terms like $\partial^{2}g_{\mathrm{km}}/\partial x^{i}\partial x^{\ell }$). This might still be numerically reasonable for the present data, but we have not done so in this article, because there appears to be no obvious application or possibility of comparison with observed results. The scalar curvature varies in magnitude and even in its sign over the RGB cube, so there is certainly not anything resembling a homogeneous space.
